# Interchangeability and optimization of heart rate methods for estimating oxygen uptake in ergometer cycling, level treadmill walking and running

**DOI:** 10.1186/s12874-022-01524-w

**Published:** 2022-02-27

**Authors:** Karin Sofia Elisabeth Olsson, Hans Rosdahl, Peter Schantz

**Affiliations:** 1 The Research Unit for Movement, Health and Environment, Department of Physical Activity and Health, The Swedish School of Sport and Health Sciences, GIH, Stockholm, Sweden; 2 The Research Unit for Movement, Health and Environment, Department of Physiology, Nutrition and Biomechanics, The Swedish School of Sport and Health Sciences, GIH, Stockholm, Sweden

**Keywords:** Heart rate method, Heart rate, Oxygen uptake, Ergometer cycling, Walking, Running

## Abstract

**Background:**

The heart rate (HR) method enables estimating oxygen uptake (V̇O_2_) in physical activities. However, there is a paucity in knowledge about the interchangeability of this method when applied to cycling, walking and running. Furthermore, with the aim of optimization, there is a need to compare different models for establishing HR-V̇O_2_ relationships.

**Methods:**

Twenty-four physically active individuals (12 males and 12 females) participated. For each participant, two models of HR-V̇O_2_ relationships were individually established in ergometer cycling, level treadmill walking and running. Model 1 consisted of five submaximal workloads, whereas model 2 included also a maximal workload. Linear regression equations were used to estimate V̇O_2_ at seven intensity levels ranging between 25 and 85% of heart rate reserve (HRR). The estimated V̇O_2_ levels were compared between the exercise modalities and models, as well as with data from a previous study.

**Results:**

A high level of resemblance in estimated V̇O_2_ was noted between running and cycling as well as between running and walking, with both model 1 and model 2. When comparing walking and cycling, the V̇O_2_ levels for given intensities of %HRR were frequently slightly higher in walking with both models (range of significant differences: 5–12%). The variations of the estimated individual V̇O_2_ values were reduced when using model 2 compared to model 1, both between and within the exercise modalities.

**Conclusion:**

The HR method is optimized by more workloads and wider ranges. This leads to overall high levels of interchangeability when HR methods are applied in ergometer cycling, level treadmill walking and running.

**Supplementary Information:**

The online version contains supplementary material available at 10.1186/s12874-022-01524-w.

## Background

Applying the heart rate (HR) method for interpreting oxygen uptake (V̇O_2_) in human physical activities can be beneficial for educational purposes, health promotion and disease prevention as well as from research perspectives. This is since our understanding of physical activities’ effects on morbidity, premature mortality and weight control as well as physical performance is largely related to intensities relative to the maximal oxygen uptake (e.g. [1, 2]), energy expenditure (e.g. [3, 4]) and MET hours (e.g. [5]).

The HR method is based on the linear relationship between HR and V̇O_2_ with increasing workload under steady state conditions, which was established already in the early twentieth century [6, 7]. Since the HR-V̇O_2_ relationships differ between individuals, especially depending on sex, age, body weight and fitness level (e.g. [8–10]), the relationships need to be determined individually. In that way, individual levels of V̇O_2_ can, in principle, be estimated by HR monitoring for a wide range of intensities (with individual exceptions at very low and very high intensities [11–13]). Given the estimated V̇O_2_ levels, the energy costs can then be calculated based on caloric coefficients ([14], p. 104).

A number of studies have used the HR method for estimating oxygen uptake in both humans [13, 15] and animals [16]. However, in order for the HR method to be used for the purposes mentioned above, it needs to be well methodologically developed. Issues of importance for enhancing this approach are: (1) reproducibility, (2) methods for establishing HR-V̇O_2_ relationships, (3) validity under various applied conditions during both prolonged constant and intermittent exercise, (4) external validity in terms of interchangeability between different dynamic exercise modalities, and (5) external validity in various groups of individuals.

It is only recently that the reproducibility of the HR method has been systematically studied. These examinations, evaluating sedentary activities and treadmill walking [17] as well as ergometer cycling [18, 19], have demonstrated a good reproducibility with minor relative mean differences between test and retest. However, at the individual level, there is a variation that prompts stabilizing the establishment of HR-V̇O_2_ relationships. This requires further investigations. Possibilities in this respect may include more workloads and wider ranges when establishing the HR-V̇O_2_ relationships.

With regard to the interchangeability of the HR method between different forms of physical exercise, it has long been known that dynamic exercise using smaller muscle groups, compared to larger, induce a higher heart rate at a given level of V̇O_2_ [20–22]. If instead focusing on various dynamic exercises with only large muscle groups involved, the question of interchangeability is still a rather unexplored research area.

For various reasons, ergometer cycling is a valuable point of departure in this respect. This is because different workloads of ergometer cycling induce known mean levels of V̇O_2_ at steady state [23–25]. This enables the HR method to be used in education as well as in health promotion and disease prevention when a high level of accuracy is not critical. Furthermore, regardless of whether the HR-V̇O_2_ relationships are established through measurements or estimations of V̇O_2_, application of the HR method in field conditions is helpful for studies using larger groups of participants. This is since mobile metabolic measurement systems are both costly and technically complicated to use, and can be sensitive to ambient conditions [26, 27].

Given this background, it would be particularly valuable if the HR method, based on ergometer cycling, is interchangeable with other common forms of dynamic exercise that involve large muscle groups, such as walking and running. To our knowledge, Olsson, Salier Eriksson [28], is the only study that has systematically examined this in relation to level walking on treadmill. Although there was a general concordance in estimated mean levels of V̇O_2_ for given percentages of heart rate reserve (%HRR), there were indications of a reduced individual variation when a maximal workload was added to three submaximal levels of exercise. Indeed, this indicated that varying the ways of establishing HR-V̇O_2_ relationships can lead to different degrees of methodological optimization. In the present study, we have therefore developed the HR method to include more submaximal workloads (five vs three), and in combination with maximal workloads and wider measurement ranges. The potential optimizing effects will be evaluated through comparisons within the results of the present study as well as with Olsson, Salier Eriksson [28]. Finally, we extend the question about interchangeability through also examining level running on treadmill.

## Methods

### Participants

Twenty-four healthy and physically active individuals (12 men and 12 women) participated in the study. Most of them were recruited among the students at the Swedish School of Sport and Health Sciences, GIH, Stockholm, Sweden. Others were recruited through various training groups of recreational runners. The inclusion criteria were: man or woman between 20 and 45 years, healthy and injury free, familiar to cycling, walking and running, and performing endurance training regularly, at least 30 min twice a week, during at least the year prior to the study. Anthropometric characteristics and resting HR values for the participants are given in Table 1. Prior to the study, they were informed about the research project and their rights as participants. This information, as well as a health declaration, were sent to them before their start in the study. At the first occasion, a written informed consent of participation was signed of all participants, and they certified themselves to be healthy for participation. An ethical approval to conduct the study was obtained from the Ethics Committee North of the Karolinska Institute at the Karolinska Hospital (Dnr 03–637), Stockholm, Sweden.

**Table 1:** Anthropometric characteristics and resting HR

Participants		Age	Height	Weight	BMI	HRrest
	(n)	(years)	(m)	(kg)	(kg·m^**−2**^)	(beats·min^**−1**^)
**Women**	**12**	29.4 ± 8.1	1.66 ± 0.07	60.0 ± 5.3	21.8 ± 1.6	49.7 ± 6.9
**Men**	**12**	28.8 ± 7.9	1.83 ± 0.09	80.8 ± 9.3	23.9 ± 1.1	51.4 ± 6.1
**All**	**24**	29.1 ± 7.8	1.75 ± 0.12	70.4 ± 12.9	22.9 ± 1.7	50.6 ± 6.5

Values are presented as mean ± standard deviation (SD)

### Study design, standardization and experimental procedures

The experimental procedures consisted of four different test occasions on the equal amount of days. The first occasion, always conducted in the morning, was a pre-test and included measurements of the resting HR and the resting metabolism (data not used). HR was collected continuously while the subject rested in supine position on a treatment table for 30 min. After this measurement, a familiarization of trying out the equipment followed through performing submaximal exercise in ergometer cycling, level treadmill walking and running. During the three subsequent occasions, the various exercise modalities were performed in a randomized order. The cycling and running occasions, included both submaximal and maximal workloads, while the walking included only submaximal workloads. In most cases, the time of the day when these three sessions were performed varied within two hours for each participant. All four test occasions were initially scheduled at 5–7 day intervals. However, due to illness for some participants, this interval became 8 ± 5 days (mean ± SD). All measurements took place in a laboratory with well-controlled ambient conditions (room temperature 18–21 °C).

The participants were instructed to keep the following standardization procedures prior to the measurements. Before all occasions, they were asked to refrain from vigorous exercise 24 h preceding the tests. They were also asked to avoid stress, to travel to the laboratory in a manner that was not physically strenuous and to cancel the tests if they had either fever, an infection or a cold. Before the first occasion, they were instructed to be fasting in the morning (only water allowed) and refrain from eating at least 8 hours before the test. Prior to the subsequent occasions, instructions were to refrain from eating, consuming coffee, tea and energy drinks, smoking and taking snuff during 1 hour before the tests. Ingestions of heavier meals were allowed at least 3 hours before the tests.

All four occasions started with measurements of body weight (without shoes, but typically with shorts and t-shirts), while the body height was only measured at the first occasion. The measurements from the first occasion were used as baseline values (Table 1). The other measurements of weight were used to describe V̇O_2_ in relation to body weight when performing the various exercise modalities (Additional file 1: Table S1-S3). At the last three occasions, a measurement of the resting HR followed in a standardized procedure, while the participant rested in supine position on a treatment table for ten minutes. The HR values during the last 5 minutes were averaged. In this way, each occasion was initiated in the same manner, and the individual day-to-day variation could been observed. These intra-individual HR variations were mainly small (absolute median difference: 3.9 beats·min^− 1^). However, for six individuals their largest differences were between 10.0–15.8 beats·min^− 1^. This variation was regarded as acceptable.

The submaximal workloads for the three various exercise modalities, were performed with continuous measurements of HR and V̇O_2_, and continued until steady state had been reached for two consecutive minutes. This was normally attained after 5 minutes. The criterion for steady state was that HR was within 2–3 beats·min^− 1^. Rating of perceived exertion (RPE), according to Borg’s RPE scale 6–20 [29], was assessed separately for legs and breathing, at the end of each submaximal workload. The maximal cycle ergometer and treadmill tests were performed until voluntary exhaustion occurred. HR and V̇O_2_ were measured continuously, while RPE for both legs and breathing, respectively, were assessed directly after the tests. In order to ensure that the maximal tests attained their purposes, at least two of the following three criteria were met by each participant: 1) a plateau in V̇O_2_ despite increasing exercise intensity (defined as a V̇O_2_ increment of < 150 ml·min^− 1^), 2) a respiratory exchange ratio of ≥1.10, and 3) a RPE rating of ≥17 [30–32].

#### Ergometer cycling

The cycle exercise included 5–6 uninterrupted submaximal workloads and a maximal test. For all participants, the first five submaximal workloads were: 50, 75, 100, 125 and 150 W. Only these five submaximal workloads have been used in the present study. However, some participants continued and finished a 6th workload, at either 175 or 200 W. The decision on whether the last workload would be 150, 175 or 200 W, was primarily due to HR and RPE at 150 W. If HR was ≥170 beats·min^− 1^ or if RPE, either legs or breathing, was ≥17, the submaximal phase was ended after 150 W. When HR was between 150 and 169 beats·min^− 1^ or that RPE, either legs or breathing, was between 15 and 16, 175 W was in general chosen as the last workload. If HR was < 150 beats·min^− 1^ and RPE, both legs and breathing, was < 15, then 200 W was in all cases chosen as the final workload. Throughout all submaximal workloads, the participants sat in an upright position with their hands laying relaxed on the handlebars, while using a cycling cadence of 50 revolutions per minute (rpm) in accordance with earlier studies [23–25]. After the submaximal exercise, approximately 10 min of rest followed before the maximal test. During the last 2 minutes of this period, the participant started to cycle at a light resistance (about 7.4 N) using a self-chosen cadence.

The maximal cycling test was carried out through three different protocols, all using a cadence of 80 rpm. Protocols 1, 2 or 3 were used if the last submaximal workload had been 150, 175 or 200 W, respectively. The starting level was 60 W in all protocols, but during the first minute, each protocol used a varying schedule. Protocol 1 kept constant resistance. The 2nd protocol increased by 40 W after the first 20 s and then by 20 W after the next 20 s. The 3rd protocol increased by 40, 20 and 40 W after each period of 15 s. After the first minute, all protocols increased by 40 W, thereafter the resistance increased by 20 W every continued minute until exhaustion ended the test.

#### Treadmill walking

Five submaximal workloads of uninterrupted level (inclination 0.0°) walking were used at the following speeds: 3, 4, 5, 6, and 7 km·h^− 1^.

#### Treadmill running

Six submaximal workloads of uninterrupted level running and a maximal test were performed. The submaximal speeds were set at: 6, 7, 8, 10, 12 and 13 or 14 km·h^− 1^. However, only the first five submaximal workloads have been used in the present study. After the submaximal workloads, about 10 min of rest followed before the maximal test. During the last few minutes of this period, the participant walked at a slow pace (about 4 km·h^− 1^) on the treadmill.

The maximal running test was performed through constant speed and successive increments of inclination every full minute. During the first minute, the inclination was set to 0.0°, after 1 minute it was increased to 1.0°, and then by 0.5° every minute until the test was ended. The constant running speed (12.7 ± 0.9 km·h^− 1^) was individually selected for each participant. The selection of speed was based on the participants’ general perceived exertion during the submaximal exercise. In agreement with each participant, an individual speed corresponding to approximately 15 on the RPE scale was chosen.

### Equipment and preparations

#### Cycle ergometer and treadmill

The cycle exercise was performed on a manually braked Monark pendulum cycle ergometer 828E (Monark Exercise AB, Vansbro, Sweden). Prior to the study, service and calibration procedures were undertaken according to the manufacturer’s advice. Immediately before each phase of submaximal as well as maximal exercise, the scale was zeroed while the participant sat on the saddle with the feet resting on the surface between the pedals. A digital metronome (DM70 Seiko S-Yard Co. Ltd., Tokyo, Japan) was used to maintain correct cycling cadence. At regular intervals of 1 minute, the work rate was controlled by checking the cadence and the braking force as indicated by the pendulum position. The treadmill exercises were carried out on a RL2500E treadmill (Rodby Innovation AB, Vänge, Hagby, Sweden). Preceding the study, both the speed and the inclination were carefully controlled.

#### Stationary metabolic system

A stationary metabolic gas analysis system with a mixing chamber, Jaeger Oxycon Pro® (Carefusion GmbH, Hoechberg, Germany), was used during all metabolic measurements. This system has been shown to be valid and reliable [27, 33]. It was switched on around 30 min preceding data collection and calibrations were made prior to each individual test occasion, according to the manufacturer’s recommendations. A high precision gas, composed of 15.00% O_2_ and 6.00% CO_2_ (accuracy: O_2_ ± 0.04% and CO_2_ ± 0.1%) (Air Liquid AB, Kungsängen, Sweden), was used for calibration. In all tests, the participants wore a face mask, ORO-NASAL (Hans Rudolph Inc., Kansas City, MO, USA) with a lightweight non-rebreathing 3-way valve (Innovision A/S, Odense, Denmark). The valve was connected to a lightweight tube, 1.8 m long, (Innovision A/S, Odense, Denmark) for leading the exhaled air into the mixing chamber.

#### Heart rate monitor

The HR measurement was performed using a Polar RS400 monitor and the associated Polar WearLink transmitter (Polar Electro, Kempele, Finland).

### Data processing and statistical analyses

All HR and V̇O_2_ values were saved in mean values of 15 s for data processing. For determination of the resting HR, the average of the last 5 minutes from the rest measurement at the first occasion was used. For all submaximal workloads, paired HR and V̇O_2_ values in the last, of the two consecutive minutes at steady state, have been used. In accordance with Howley, Bassett [31], the maximal HR and V̇O_2_ values were calculated by averaging the minute with highest continuous paired values. In the calculation of percentages of heart rate reserve (%HRR) during exercise, both the resting and maximal values were used in the equation; ((“exercise HR” - HRrest) · (HRmax - HRrest)^− 1^) · 100. Furthermore, the calculated relative exercise intensities for ergometer cycling, in terms of HRmax, HRR and V̇O_2_max, have been based on the maximal cycling values. Conversely, the values from the maximal treadmill test were used to describe the relative intensities for both walking and running.

For application of the HR method, two different models of HR-V̇O_2_ relationships were individually established for each of the three exercise modalities. These relationships were based on paired HR and V̇O_2_ values from five submaximal workloads (model 1), plus one maximal workload (model 2), and were calculated as linear regression equations. The HR-V̇O_2_ relationship in model 2 for cycling, was based on the maximal cycling values, while the HR-V̇O_2_ relationships for walking and running, respectively, were based on the values from the maximal treadmill test. The individual regression equations were then applied as parts of HR methods for estimating V̇O_2_ using individually derived HR values based on seven levels of %HRR according to the American College of Sports Medicine (ACSM) classification of exercise intensity [34]. The intensity levels, 25 and 35%HRR (very light to light), 45 and 55%HRR (moderate), and 65, 75 and 85%HRR (vigorous), were selected to cover a wide and commonly used intensity range for physical activity. For this determination of individual HR values, in terms of %HRR, the maximal HR values from the treadmill test were always used.

Exercise mode differences were calculated in both absolute and relative terms for the constituents of regression equations (y-intercept, slope and r^2^) and the V̇O_2_ estimations in both model 1 and model 2. Confidence intervals (CI) of 95% were calculated for all these differences as well as the absolute values. In the calculations of the relative differences, cycling was used as the reference value in the comparisons with walking and running, while walking was used as the basis when comparing with running. The absolute and relative exercise mode differences were evaluated jointly for all participants with the one-sample t-test, since no systematic variations were seen between the sexes when analysed with an independent t-test. A Bonferroni post-hoc test ([35], p. 377) was applied since all values in the exercise mode comparisons were analysed twice. Instead of dividing the level of significance with the number of comparisons, the *P*-values obtained were multiplied by two. This allowed *P* < 0.05 to be used as the significance level.

Linear regression lines, based on all participants individually estimated V̇O_2_ values between 25 and 85%HRR, were used to illustrate mean V̇O_2_ for all exercise modalities in both models. The corresponding regression equations were calculated. All participants’ individually estimated V̇O_2_ values were also graphically pairwise compared between all exercise modalities and models in scatter plots, and with linear regressions equations. The statistical analyses have been performed using the Statistical Package for the Social Sciences (IBM SPSS Statistics, 25 and 26, Chicago, IL, USA). Figures were created in GraphPad Prism® 8 software package (GraphPad Software Inc., San Diego, CA, USA). Values are given as mean ± SD, unless otherwise stated.

## Results

### Measurement positions for the establishments of HR-V̇O_2_ relationships

**Fig. 1:**
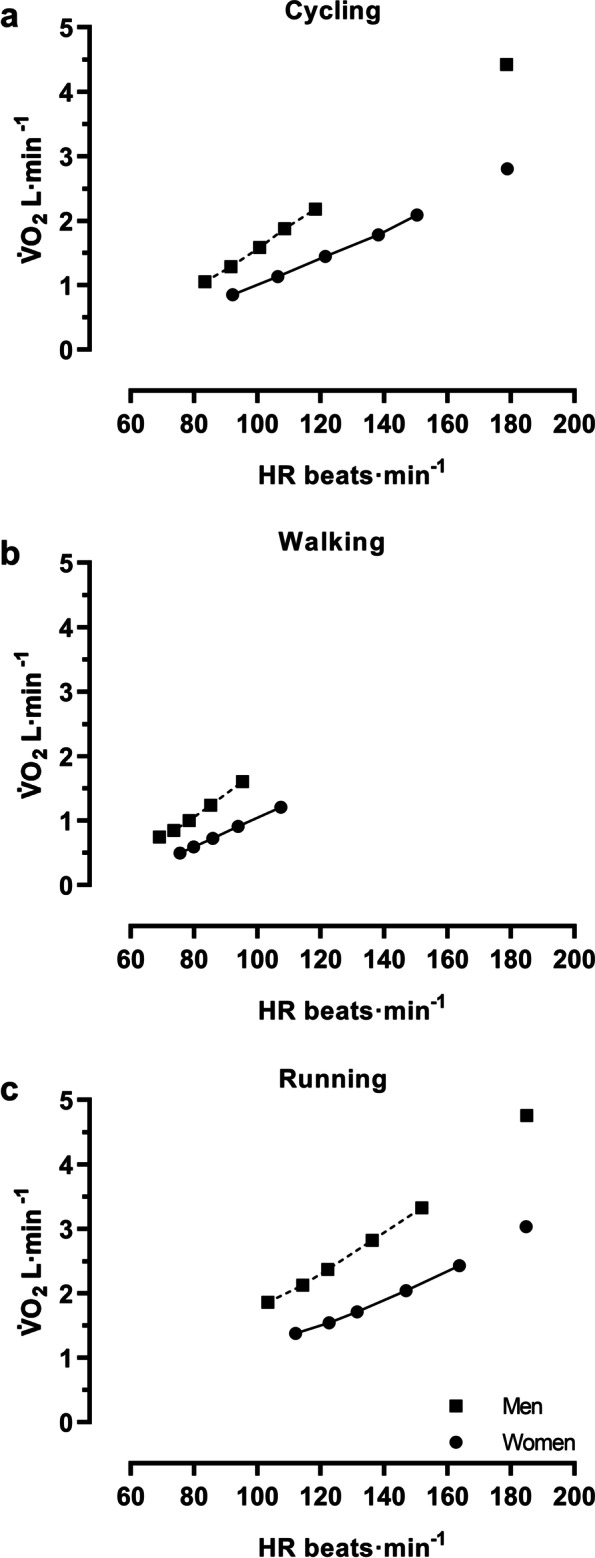
Average measurement positions for the establishments of individual HR-V̇O_2_ relationships. Five submaximal positions (symbols with connected lines) were used to establish the HR-V̇O_2_ relationships for both model 1 and model 2, whereas maximal positions (separated symbols) were used only for model 2. The figure is based on the individual values of all males (*n* = 12) and all females (n = 12), separately. **a** Cycling, **b** walking, and **c** running

The measurement positions from the submaximal and maximal workloads that were used to establish the individual HR-V̇O_2_ relationships for ergometer cycling, treadmill walking and running are illustrated in Fig. 1 as mean values for males and females, separately. For further details on the workloads performed, such as relative exercise intensities of HR and V̇O_2_ as well as RPE levels, see Additional file 1 (Table S1-S3).

### Regression equations

For application of the HR method, individual linear regression equations were created for both model 1 (based on only submaximal workloads) and model 2 (based on both submaximal and maximal workloads) for each of the three exercise modalities. When studying the absolute and relative exercise mode differences of all participants with both models, there were only three exercise mode differences in y-intercepts and slopes. With regard to the r^2^-coefficients, no mode differences were noted in model 1, while in model 2 all differences were significant, but of a minor degree. Both the absolute values of the regression coefficients as well as the modality comparisons can be found in Additional file 2 (Table S4 and S5).

### Application of HR methods

#### Interchangeability between the exercise modalities

The estimated mean V̇O_2_ levels, when applying the HR method at 25–85%HRR for ergometer cycling, treadmill walking and running are presented for model 1 in Table 2 and for model 2 in Table 3. Absolute and relative V̇O_2_ differences between the exercise modalities are given in pairwise comparisons as measures of the interchangeability.

**Table 2:** Estimation of V̇O_2_ with model 1 and exercise mode comparisons

Exercise intensity								
		Very light – light		Moderate		Vigorous		
		25%HRR	35%HRR	45%HRR	55%HRR	65%HRR	75%HRR	85%HRR
**HR (beats·min**^**− 1**^**)**		84.1 ± 6.2	97.6 ± 6.3	111.0 ± 6.5	124.4 ± 6.8	137.9 ± 7.2	151.3 ± 7.7	164.7 ± 8.2
**Estimation of**	**Cycling**^**a**^	0.88 ± 0.31	1.25 ± 0.40	1.62 ± 0.49	1.98 ± 0.59	2.35 ± 0.69	2.72 ± 0.79	3.09 ± 0.89
**V̇O**_**2**_ **(L·min**^**− 1**^**)**		(0.75 to 1.01)	(1.08 to 1.42)	(1.41 to 1.82)	(1.74 to 2.23)	(2.06 to 2.64)	(2.39 to 3.05)	(2.71 to 3.46)
	**Walking**^**b**^	0.96 ± 0.35	1.34 ± 0.42	1.72 ± 0.50	2.10 ± 0.58	2.48 ± 0.67	2.86 ± 0.76	3.25 ± 0.85
		(0.81 to 1.10)	(1.16 to 1.51)	(1.51 to 1.93)	(1.86 to 2.35)	(2.20 to 2.77)	(2.54 to 3.19)	(2.89 to 3.61)
	**Running**^**c**^	1.00 ± 0.35	1.34 ± 0.40	1.69 ± 0.46	2.03 ± 0.52	2.37 ± 0.58	2.72 ± 0.65	3.06 ± 0.72
		(0.85 to 1.15)	(1.17 to 1.51)	(1.49 to 1.88)	(1.81 to 2.25)	(2.13 to 2.62)	(2.44 to 2.99)	(2.76 to 3.36)
**Absolute**	**Walk vs Cyc**	0.08 ± 0.19	0.09 ± 0.19	0.10 ± 0.22	0.12 ± 0.26	0.13 ± 0.31	0.14 ± 0.37	0.16 ± 0.43
**differences**		(− 0.00 to 0.16)	(0.01 to 0.17)	(0.01 to 0.20)	(0.01 to 0.23)	(− 0.00 to 0.26)	(− 0.01 to 0.30)	(− 0.02 to 0.34)
**(L·min**^**− 1**^**)**	**Run vs Cyc**	0.12 ± 0.18**	0.10 ± 0.15*	0.07 ± 0.15	0.04 ± 0.18	0.02 ± 0.22	− 0.01 ± 0.27	− 0.03 ± 0.33
		(0.05 to 0.20)	(0.03 to 0.16)	(0.01 to 0.13)	(− 0.03 to 0.12)	(− 0.07 to 0.11)	(− 0.12 to 0.11)	(− 0.17 to 0.11)
	**Run vs Walk**	0.04 ± 0.21	0.00 ± 0.18	− 0.03 ± 0.17	− 0.07 ± 0.20	−0.11 ± 0.25	−0.15 ± 0.32	−0.19 ± 0.38
		(−0.05 to 0.13)	(− 0.07 to 0.08)	(− 0.11 to 0.04)	(− 0.16 to 0.01)	(− 0.22 to − 0.00)	(− 0.28 to − 0.02)	(−0.35 to − 0.03)
**Relative**	**Walk vs Cyc**	11.5 ± 29.0	8.6 ± 18.0	7.5 ± 14.4*	6.9 ± 13.1*	6.6 ± 12.8*	6.3 ± 12.8*	6.2 ± 12.9
**differences**		(−0.7 to 23.7)	(1.0 to 16.2)	(1.5 to 13.6)	(1.4 to 12.5)	(1.2 to 12.0)	(0.9 to 11.7)	(0.7 to 11.6)
**(%)**	**Run vs Cyc**	15.0 ± 21.9**	8.7 ± 13.3**	5.4 ± 9.9*	3.4 ± 8.5	2.1 ± 8.2	1.1 ± 8.2	0.4 ± 8.4
		(5.8 to 24.3)	(3.1 to 14.3)	(1.3 to 9.6)	(−0.2 to 7.0)	(− 1.4 to 5.5)	(− 2.3 to 4.6)	(−3.2 to 4.0)
	**Run vs Walk**	7.9 ± 28.7	1.7 ± 15.1	− 0.9 ± 11.0	−2.4 ± 10.0	−3.3 ± 10.2	−3.9 ± 10.8	− 4.4 ± 11.4
		(− 4.2 to 20.1)	(−4.7 to 8.1)	(− 5.6 to 3.7)	(−6.6 to 1.9)	(− 7.6 to 1.0)	(− 8.5 to 0.6)	(− 9.2 to 0.4)

Values are based on all participants’ (*n* = 24) individual regression equations (Additional file 2: Table S4) and presented as mean ± SD and (95% CI).

Significance of exercise mode differences: *P < 0.05, **P < 0.01

Calculation of the absolute differences: Walk vs Cyc = b-a, Run vs Cyc = c-a, and Walk vs Run = c-b.

Calculation of the relative differences: Walk vs Cyc = ((b-a) · a^− 1^) · 100, Run vs Cyc = ((c-a) · a^− 1^) · 100, and Walk vs Run = ((c-b) · b^− 1^) · 100.

**Table 3:** Estimation of V̇O_2_ with model 2 and exercise mode comparisons

Exercise intensity								
		Very light – light		Moderate		Vigorous		
		25%HRR	35%HRR	45%HRR	55%HRR	65%HRR	75%HRR	85%HRR
**HR (beats·min**^**−1**^**)**		84.1 ± 6.2	97.6 ± 6.3	111.0 ± 6.5	124.4 ± 6.8	137.9 ± 7.2	151.3 ± 7.7	164.7 ± 8.2
**Estimation of**	**Cycling**^**a**^	0.84 ± 0.35	1.23 ± 0.41	1.61 ± 0.49	2.00 ± 0.58	2.39 ± 0.67	2.78 ± 0.76	3.17 ± 0.85
**V̇O**_**2**_ **(L·min**^**− 1**^**)**		(0.69 to 0.98)	(1.05 to 1.40)	(1.41 to 1.82)	(1.76 to 2.25)	(2.11 to 2.67)	(2.46 to 3.10)	(2.81 to 3.53)
	**Walking**^**b**^	0.95 ± 0.37	1.34 ± 0.44	1.73 ± 0.51	2.12 ± 0.59	2.52 ± 0.67	2.91 ± 0.76	3.30 ± 0.84
		(0.80 to 1.11)	(1.16 to 1.53)	(1.52 to 1.95)	(1.87 to 2.38)	(2.23 to 2.80)	(2.59 to 3.23)	(2.94 to 3.66)
	**Running**^**c**^	0.86 ± 0.34	1.25 ± 0.39	1.64 ± 0.46	2.03 ± 0.54	2.42 ± 0.62	2.81 ± 0.71	3.21 ± 0.79
		(0.72 to 1.00)	(1.08 to 1.41)	(1.45 to 1.83)	(1.81 to 2.26)	(2.16 to 2.69)	(2.52 to 3.11)	(2.87 to 3.54)
**Absolute**	**Walk vs Cyc**	0.11 ± 0.23	0.12 ± 0.21*	0.12 ± 0.19*	0.12 ± 0.17**	0.12 ± 0.17**	0.13 ± 0.17**	0.13 ± 0.18**
**differences**		(0.02 to 0.21)	(0.03 to 0.20)	(0.04 to 0.20)	(0.05 to 0.19)	(0.05 to 0.19)	(0.06 to 0.20)	(0.05 to 0.20)
**(L·min**^**−1**^**)**	**Run vs Cyc**	0.02 ± 0.20	0.02 ± 0.17	0.03 ± 0.15	0.03 ± 0.14	0.03 ± 0.13	0.03 ± 0.14	0.04 ± 0.16
		(− 0.06 to 0.11)	(− 0.05 to 0.10)	(− 0.04 to 0.09)	(− 0.03 to 0.09)	(− 0.03 to 0.09)	(− 0.03 to 0.09)	(− 0.03 to 0.10)
	**Run vs Walk**	− 0.09 ± 0.27	− 0.09 ± 0.23	−0.09 ± 0.20	−0.09 ± 0.17*	−0.09 ± 0.14**	−0.09 ± 0.12**	−0.09 ± 0.09***
		(−0.21 to 0.02)	(− 0.19 to 0.01)	(− 0.18 to − 0.01)	(−0.17 to − 0.02)	(−0.15 to − 0.03)	(−0.14 to − 0.04)	(−0.13 to − 0.05)
**Relative**	**Walk vs Cyc**	22.6 ± 52.2	11.8 ± 23.3*	8.4 ± 14.3*	6.7 ± 10.1**	5.7 ± 7.9**	5.0 ± 6.7**	4.6 ± 6.1**
**differences**		(0.5 to 44.6)	(1.9 to 21.6)	(2.3 to 14.4)	(2.4 to 11.0)	(2.4 to 9.0)	(2.2 to 7.9)	(2.0 to 7.1)
**(%)**	**Run vs Cyc**	6.5 ± 27.6	3.5 ± 15.0	2.7 ± 9.9	2.3 ± 7.4	2.0 ± 6.1	1.9 ± 5.6	1.8 ± 5.4
		(−5.2 to 18.1)	(−2.8 to 9.9)	(− 1.5 to 6.9)	(− 0.9 to 5.4)	(− 0.6 to 4.6)	(− 0.5 to 4.2)	(−0.5 to 4.1)
	**Run vs Walk**	− 5.3 ± 29.9	− 4.9 ± 17.9	−4.2 ± 11.8	− 3.7 ± 8.0	−3.2 ± 5.5*	− 2.9 ± 3.8**	−2.6 ± 2.5***
		(− 17.9 to 7.3)	(− 12.5 to 2.7)	(− 9.2 to 0.7)	(− 7.1 to − 0.3)	(− 5.6 to − 0.9)	(− 4.5 to − 1.3)	(− 3.7 to − 1.5)

Values are based on all participants’ (*n* = 24) individual regression equations (Additional file 2: Table S5) and presented as mean ± SD and (95% CI)

Significance of exercise mode differences: *P < 0.05, **P < 0.01, ***P < 0.001

Calculation of the absolute differences: Walk vs Cyc = b-a, Run vs Cyc = c-a, and Walk vs Run = c-b

Calculation of the relative differences: Walk vs Cyc = ((b-a) · a^− 1^) · 100, Run vs Cyc = ((c-a) · a^− 1^) · 100, and Walk vs Run = ((c-b) · b^− 1^) · 100

In the comparison of walking vs cycling, the V̇O_2_ levels were frequently slightly higher for walking in both models. Significant relative differences of 6.3–7.5% were noted at 45–75%HRR in model 1 (Table 2), whereas differences of 4.6–11.8% were noted between 35 and 85%HRR in model 2 (Table 3).

Fewer, and mostly minor, but significant differences were noted in the comparisons of running vs cycling as well as of running vs walking. The V̇O_2_ levels were 5.4–15.0% higher for running as compared to cycling at 25–45%HRR in model 1 (Table 2). In model 2, the V̇O_2_ levels were 2.6–3.2% lower for running compared to walking at 65–85%HRR (Table 3).

**Fig. 2:**
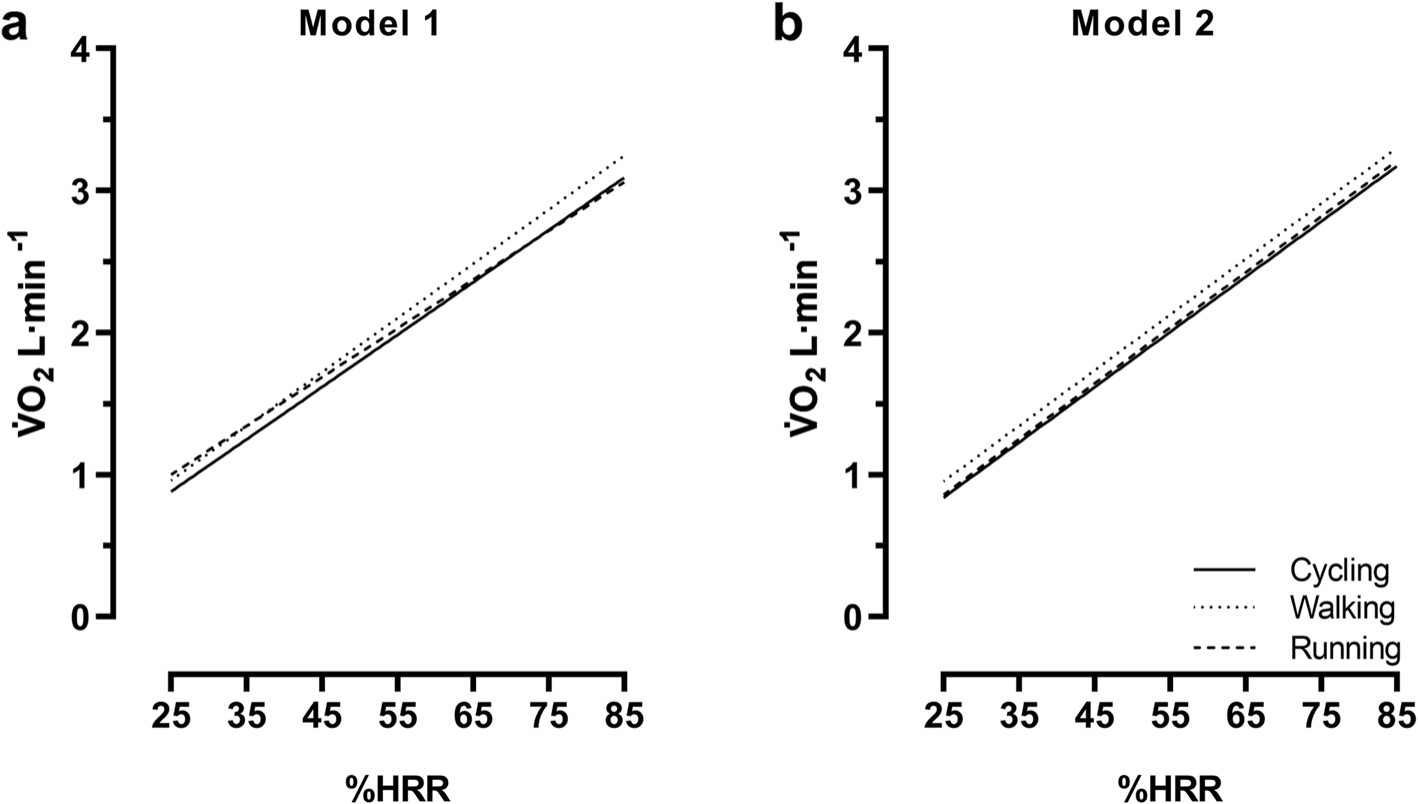
Linear regression lines for the estimated V̇O_2_. The figure is based on the individual V̇O_2_ values of all participants (n = 24) and the seven intensity levels between 25 and 85%HRR. (**a**) Model 1 and (**b**) model 2. For regression equations and r^2^-coefficients, see Table 4

Illustrations of these estimated mean V̇O_2_ levels at 25–85%HRR are given in linear regression lines in Fig. 2, and are further described through regression equations (Table 4).

**Table 4:** Linear regression equations for the estimated V̇O_2_

	y-intercept (95% CI)	slope (95% CI)	r^**2**^
Model 1: Cycling	−0.042 (− 0.316 to 0.231)	0.0368 (0.0322 to 0.0415)	0.594
Model 1: Walking	0.004 (−0.265 to 0.274)	0.0381 (0.0335 to 0.0427)	0.617
Model 1: Running	0.141 (−0.096 to 0.378)	0.0343 (0.0303 to 0.0384)	0.628
Model 2: Cycling	−0.135 (− 0.403 to 0.133)	0.0389 (0.0343 to 0.0435)	0.629
Model 2: Walking	−0.027 (− 0.299 to 0.244)	0.0391 (0.0345 to 0.0438)	0.625
Model 2: Running	−0.120 (− 0.370 to 0.130)	0.0391 (0.0349 to 0.0434)	0.663

The linear regression equations are based on the individual V̇O_2_ values of all participants (*n* = 24) and the seven intensity levels between 25 and 85%HRR (cf. Fig. 2)

#### Optimization of interchangeability between the exercise modalities

**Fig. 3:**
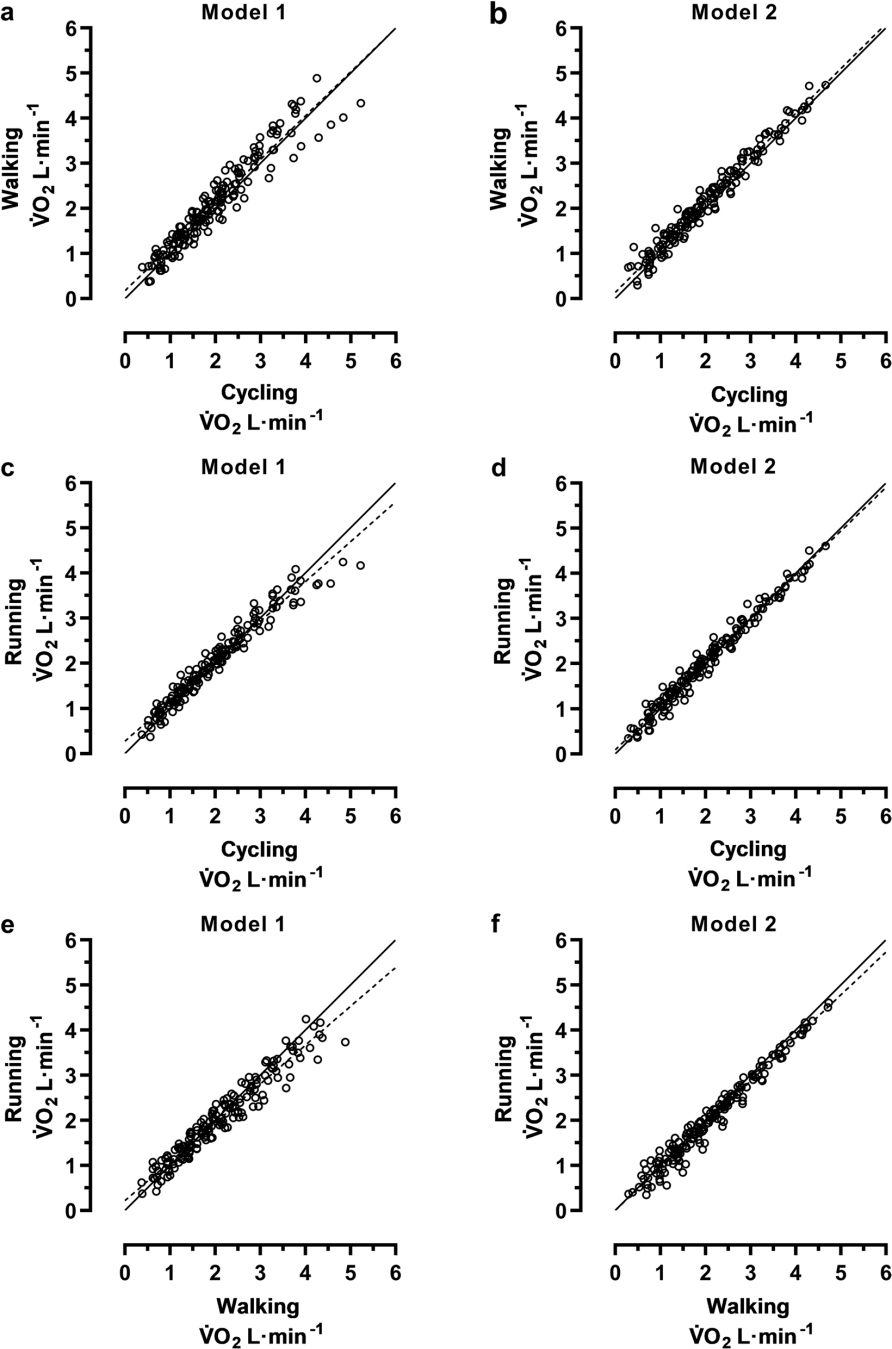
Exercise mode comparisons for the estimated individual V̇O_2_ values. The figure is based on the individual V̇O_2_ values of all participants (n = 24), ranging between 25 and 85%HRR. Line of identity = solid line and the linear regression = dashed line. Walking vs cycling; (**a**) model 1 and (**b**) model 2, running vs cycling; (**c**) model 1 and (**d**) model 2, and running vs walking; (**e**) model 1 and (**f**) model 2. For regression equations and r^2^-coefficients, see Table 5

Pairwise exercise mode comparisons of the estimated individual V̇O_2_ values are illustrated in Fig. 3, and described with linear regression equations (Table 5). In all three modality comparisons, the visually observed spreading of values was smaller in model 2 compared to model 1 (Fig. 3). Furthermore, numerically; (1) higher r^2^-coefficients, (2) y-intercepts closer to y = 0, (3) slopes closer to the lines of identity, and (4) narrower 95% CI for both y-intercepts and slopes, were noted in model 2 compared to model 1 (Table 5).

**Table 5:** Linear regression equations for the exercise mode comparisons of the estimated individual V̇O_2_ values

	y-intercept (95% CI)	slope (95% CI)	r^**2**^
Model 1: Walk & Cyc	0.178 (0.077 to 0.279)	0.970 (0.924 to 1.016)	0.913
Model 2: Walk & Cyc	0.138 (0.073 to 0.202)	0.992 (0.963 to 1.021)	0.965
Model 1: Run & Cyc	0.276 (0.209 to 0.344)	0.883 (0.853 to 0.914)	0.952
Model 2: Run & Cyc	0.093 (0.040 to 0.145)	0.968 (0.944 to 0.992)	0.975
Model 1: Run & Walk	0.218 (0.136 to 0.300)	0.862 (0.826 to 0.897)	0.933
Model 2: Run & Walk	0.004 (−0.060 to 0.067)	0.955 (0.928 to 0.982)	0.967

The linear regression equations are based on the individual V̇O_2_ values of all participants (n = 24), ranging between 25 and 85%HRR (cf. Figure 3). Walk = walking, Cyc = cycling, and Run = running

#### Optimization within each exercise modality

**Fig. 4:**
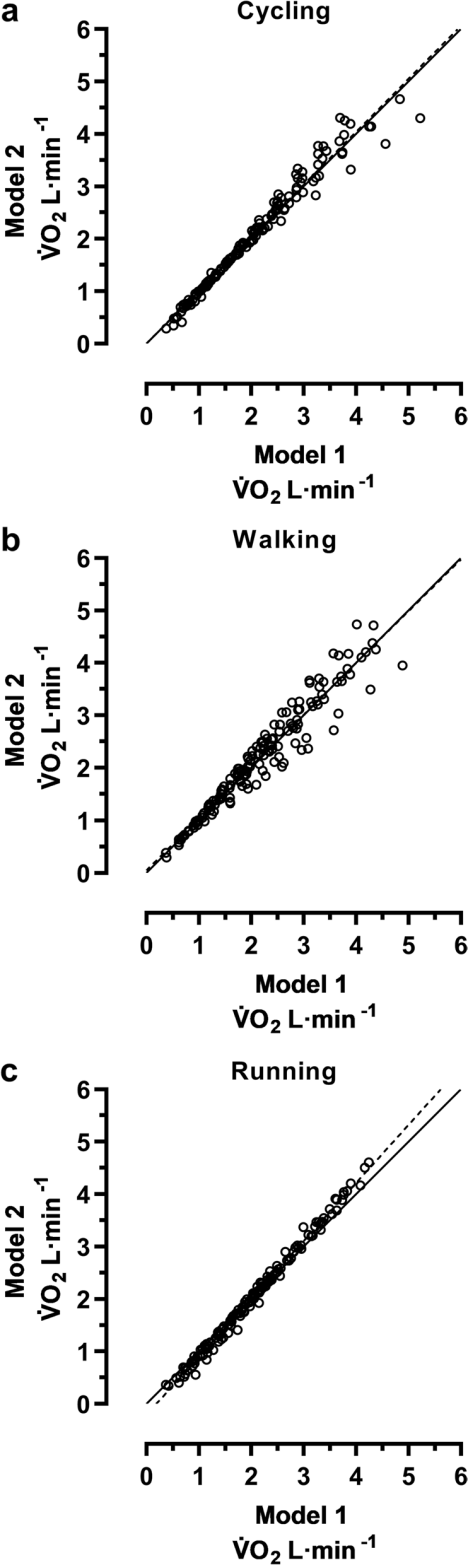
Model comparisons for the estimated individual V̇O_2_ values. The figure is based on the individual V̇O_2_ values of all participants (n = 24), ranging between 25 and 85%HRR. Line of identity = solid line and the linear regression = dashed line. (**a**) Cycling, (**b**) walking, and (**c**) running. For regression equations and r^2^-coefficients, see Table 6

When comparing the two models within each of the three exercise modalities, it is notable that the r^2^-coefficients of the linear relationships between %HRR and V̇O_2_ were somewhat numerically higher for all modalities in model 2 compared to model 1 (Table 4). At the same time, the model comparisons of individual V̇O_2_ values within each exercise modality, separately, illustrate overall minor individual variations (Fig. 4). This is also indicated by the corresponding r^2^-coefficients (range: 0.937–0.993) as well as by the y-intercepts and slopes with their 95% CI, which included or were close to the lines of identity (Table 6).

**Table 6:** Linear regression equations for the model comparisons of the estimated individual V̇O_2_ values

	y-intercept (95% CI)	slope (95% CI)	r^**2**^
Cycling: Model 1 & 2	0.000 (−0.060 to 0.060)	1.010 (0.983 to 1.037)	0.970
Walking: Model 1 & 2	0.050 (−0.041 to 0.141)	0.987 (0.948 to 1.026)	0.937
Running: Model 1 & 2	−0.212 (− 0.243 to − 0.180)	1.106 (1.092 to 1.120)	0.993

The linear regression equations are based on the individual V̇O_2_ values of all participants (n = 24), ranging between 25 and 85%HRR (cf. Fig. 4)

## Discussion

The studied interchangeability of HR methods when estimating oxygen uptake for ergometer cycling, level treadmill walking and running was undertaken at submaximal levels between 25 and 85% of HRR, according to the ACSM classification of exercise intensities [34]. An overall good interchangeability of the HR methods was noted between the various exercise modalities, although the V̇O_2_ levels for walking were frequently slightly higher (5–12%) compared to cycling.

Another part of this study evaluated if various forms of establishing HR-V̇O_2_ relationships can optimize HR methods. For this purpose, two different models of HR-V̇O_2_ relationships were constructed. Model 1 used five submaximal workloads, whereas model 2 also included a maximal workload. With this aim of optimization, systematic comparisons have also been made between the present study and Olsson, Salier Eriksson [28] that used only three submaximal workloads to establish the HR-V̇O_2_ relationships (for more details, see Additional file 3). The overall results from these analyses indicate that the inclusions of more workloads (both submaximal and maximal), and in combination with wider measurement ranges, reduce the individual variations when using HR methods. This increased stability in the HR-V̇O_2_ relationships makes the HR methods more accurate. Note, however, that these analyses of optimization strategies are based on pairwise numerical comparisons. Due to the low number (two or three) of possible comparisons, there has been no basis for statistical analyses. Moreover, it cannot be ruled out that the differences observed between the two studies (cf. Additional file 3) may, at least partly, be due to that various groups of participants have been used. Therefore, it would be valuable to also evaluate issues of optimization through intra-individual comparisons.

### Interchangeability between the exercise modalities

The interchangeability between running and cycling as well as running and walking was essentially good in both models. This is in line with our interpretations ([28], S1 Discussion) of earlier studies [36, 37]. Although, the comparison of running and cycling in model 1 demonstrated three rather high relative differences (range: 5.4–15.0%), they were located at low exercise intensities (25–45%HRR) (Table 2), and are therefore of no practical importance as they refer to abnormally used intensities for running. However, since the HR method also enables estimations from running to cycling, caution should be considered at the lower intensities.

In the comparisons of walking and cycling, slightly higher mean V̇O_2_ levels were frequently noted for walking in both models (model 1: 6.3–7.5%; model 2: 4.6–11.8%) (Tables 2 and 3). Therefore, these differences can be compensated in an application of HR methods. In our previous study, only two small differences were noted (3.7 and 4.4%) [28]. The different outcomes are probably mainly due to the reduction of individual variations within the HR methods of this study (see Additional file 3). Support for the present results comes from linear regression analyses, performed by us, of mean HR and V̇O_2_ data in an earlier study by Grimby and Söderholm [38]. In general, higher V̇O_2_ estimations (on average 8%) for level treadmill walking compared to ergometer cycling were noted when HR methods were applied at four fixed HR values between 100 and 145 beats·min^− 1^. These overall results contradict our previous interpretations ([28], S1 Discussion) which indicated that only HR methods based on treadmill exercise using inclination overestimate V̇O_2_ compared to ergometer cycling. Instead, this might also be the case for level treadmill walking.

Possible reasons for the deviations in oxygen pulse (V̇O_2_/HR) for level treadmill walking compared to ergometer cycling could be a slightly modified arteriovenous oxygen difference or a modified stroke volume due to a different peripheral resistance or end-diastolic volume. A tentative explanation for why it is walking that these deviations are observed for, can be related to differences in exercise intensity levels. This is since it has long been known that the stroke volume increases gradually from low intensities up to about 40–50% of V̇O_2_max during exercise [39], and in some cases it continues to increase slightly over the full range up to maximal intensity [40, 41]. When studying the present submaximal intensity ranges in relation to V̇O_2_max for all participants, it is interesting to note that the average range for walking (16–37%) never exceeds 40% of V̇O_2_max, and it is of a clearly lower intensity spectrum compared to both cycling (27–62%) and running (42–75%) (Additional file 1: Table S1-S3). This indicates that the different intensity ranges may play a role in the deviations in oxygen pulse for walking. On the other hand, the arteriovenous oxygen difference has also been shown to increase more rapidly between low intensities and up to about 40% of V̇O_2_max than compared to higher relative intensities [39, 42]. This means that the increase in arteriovenous oxygen difference, combined with the increased stroke volume, may lead to similar increases in oxygen pulse at lower and higher exercise intensities. Still, minor differences in these relations might remain, and might thereby explain the small differences noted between walking and cycling.

### Optimization of interchangeability between the exercise modalities

In all exercise mode comparisons of individual V̇O_2_ values, a reduced individual variation was observed when the submaximal HR-V̇O_2_ relationships (model 1) were extended with a maximal workload (model 2) (Table 5; Fig. 3). These indications of stabilization, from model 1 to model 2, were also noted in Olsson, Salier Eriksson [28].

A reduction of individual V̇O_2_ variation was also observed when comparing the use of three submaximal workloads in Olsson, Salier Eriksson [28] with the present use of five submaximal points and a wider range for walking (Additional file 3: Table S6; Fig. S2 and S3). Thus, it seems that an extension of the measurement ranges, through inclusion of additional submaximal and/or a maximal point, reduces the individual variations. This in turn optimizes the HR method by becoming more accurate when evaluating results at the group level, and as a likely consequence, more significant exercise mode differences were detected between walking and cycling in the present study compared to Olsson, Salier Eriksson [28].

### Optimization within each exercise modality

The HR method can also be optimized within each exercise modality. This was mirrored in the linear relationships between %HRR and V̇O_2_ through somewhat numerically higher r^2^-coefficients in model 2 compared to model 1 (Table 4). The spreadings and the regression coefficients of the model comparisons of the individual V̇O_2_ values within each exercise modality (Table 6; Fig. 4), confirm that there are only small variations between the two models. From that perspective, the submaximal relationships (model 1) are sufficient when using HR methods within the same form of exercise as they are established.

When comparing the present results of the model comparisons of individual V̇O_2_ values in walking with Olsson, Salier Eriksson [28], that used fewer submaximal workloads and a narrower range, the individual variation was clearly reduced (Additional file 3: Table S7; Fig. S4b and S5b). The corresponding analyses for cycling showed already a low degree of individual variation in the previous study [28], and it did not become further reduced in the present study (Additional file 3: Table S7; Fig. S4a and S5a). We interpret that the different outcomes in cycling and walking, respectively, are primarily dependent on the differences in widths of the measurement ranges (see Additional file 3: Fig. S1).

### External validity

The present results need to be viewed from a perspective of external validity. First of all, the participants examined were mostly students in physical education and health, and trained aerobically (V̇O_2_max running: 59.1 and 50.4 mL · min^− 1^ · kg^− 1^ in males and females, respectively) (Additional file 1: Table S3). Whether the present results are applicable to other groups of participants (e.g. older or less trained individuals) need to be evaluated. However, notably, the findings in our previous study [28], based on a group of middle-aged active commuters, are in several ways similar to the present study. This indicates that the findings might be reproducible in physically active individuals with different ages.

Furthermore, all data collection was conducted under standardized conditions in a laboratory. A natural consequential question is therefore whether the present results can be applied under field conditions. Indications that this is possible are demonstrated in a recently published study [43]. It showed that HR methods, based on ergometer cycling in a laboratory, can be valid at a group level for estimating intensity spectrums of V̇O_2_ during cycle commuting in field. On the other hand, it is currently unexplored whether HR methods’ interchangeability between different exercise modalities in laboratory conditions are applicable in field exercises. For future application of the HR method, further studies of these issues of external validity are therefore suggested.

### Practical application

Although further studies on the external validity are recommended, the results of the present study should be interpreted as a basis for future research in exercise physiology. The new added values of interchangeability and methodological optimization strategies make an already well-established method more adaptable and accurate, and therefore, a powerful alternative to other tools for measuring physical responses of human activities. In addition, the present results facilitate applications of HR methods in contexts of health education and promotion as well as disease prevention. This is since our findings allow HR methods to be individually established on a cycle ergometer, and then applied through estimating V̇O_2_ during different physical activities in field with only HR measurements.

A final aspect that is worth recalling is that the present HR methods were applied to estimate V̇O_2_ at a wide range of exercise intensity levels (25–85% of HRR). It is important to note that the V̇O_2_ values obtained are based on calculations from measured values, but, theoretically estimated for the three different exercise modalities. This means that estimations of V̇O_2_ levels, in the cases of walking and running, have been made that are not practically applicable, i.e. at high and low V̇O_2_ levels, respectively. However, with regard to the present aim of interchangeability, these results are still valuable since the HR method can be applied from both directions, e.g. from walking to running, and vice versa.

## Conclusion

This study has demonstrated an overall good interchangeability of HR methods when estimating V̇O_2_ for ergometer cycling, level treadmill walking and running, at mean levels ranging between 25 and 85% of HRR. However, the V̇O_2_ levels for walking were frequently slightly higher (about 5–12%) as compared to cycling, which can be compensated for as a part of the HR method. Finally, both the use of additional submaximal workloads (five vs three) over wider ranges as well as the inclusion of maximal workloads, generally indicated a stabilizing effect on the HR-V̇O_2_ relationships, thereby increasing the interchangeability, and optimizing the HR methods.

## Supplementary Information

**Additional file 1: Table S1** Measured V̇O_2_, HR, V̇O_2_/HR and RPE for submaximal and maximal ergometer cycling. **Table S2** Measured V̇O_2_, HR, V̇O_2_/HR and RPE for submaximal treadmill walking. **Table S3** Measured V̇O_2_, HR, V̇O_2_/HR and RPE for submaximal and maximal treadmill running.

**Additional file 2: Table S4** Regression equations of model 1 and exercise mode comparisons. **Table S5** Regression equations of model 2 and exercise mode comparisons.

**Additional file 3: **A comparative analysis with the aim of optimizing the HR method: **Table S6** Linear regression equations for the exercise mode comparisons of the estimated individual V̇O_2_ values in Olsson, Salier Eriksson (1) and the present study. **Table S7** Linear regression equations for the model comparisons of the estimated individual V̇O_2_ values in Olsson, Salier Eriksson (1) and the present study. **Figure S1.** The average HR-V̇O_2_ measurement ranges in Olsson, Salier Eriksson (1) and the present study. **Figure S2.** Exercise mode comparisons for the estimated individual V̇O_2_ values in Olsson, Salier Eriksson (1). **Figure S3.** Exercise mode comparisons for the estimated individual V̇O_2_ values in the present study. **Figure S4.** Model comparisons for the estimated individual V̇O_2_ values in Olsson, Salier Eriksson (1). **Figure S5.** Model comparisons for the estimated individual V̇O_2_ values in the present study.

## Data Availability

The datasets used and/or analysed during the current study, and the supplementary materials, are available from the corresponding author on reasonable request.

## Abbreviations

BMI: Body mass index
CO_2_: Carbon dioxide
HR: Heart rate
HRR: Heart rate reserve
MET: Metabolic equivalent of task
O_2_: Oxygen
RPE: Rated perceived exertion
RPM: Revolutions per minute
V̇O_2_: Volume of oxygen uptake per minute
V̇O_2_/HR: Oxygen pulse
