# Supplementary material for: Interchangeability and optimization of heart rate methods for estimating oxygen uptake in ergometer cycling, level treadmill walking and running
Source: BMC Med Res Methodol. 2022 Feb 27;22:55. doi: 10.1186/s12874-022-01524-w (PMC8883654; doi:10.1186/s12874-022-01524-w)
Supplement: Supplementary file 1 — Additional file 1: Table S1 Measured V̇O2, HR, V̇O2/HR and RPE for submaximal and maximal ergometer cycling. Table S2 Measured V̇O2, HR, V̇O2/HR and RPE for submaximal treadmill walking. Table S3 Measured V̇O2, HR, V̇O2/HR and RPE for submaximal and maximal treadmill running. [file 12874_2022_1524_MOESM1_ESM.pdf]

Additional file 1

To:
Interchangeability and optimization of heart rate methods for estimating oxygen uptake in ergometer cycling, level treadmill walking and running

BMC Medical Research Methodology

Karin Sofia Elisabeth Olsson[1], Hans Rosdahl[2] & Peter Schantz[1]*

[1]The Research Unit for Movement, Health and Environment, Department of Physical Activity and Health, The Swedish School of Sport and Health Sciences, GIH, Stockholm, Sweden, [2]The Research Unit for Movement, Health and Environment, Department of Physiology, Nutrition and Biomechanics, The Swedish School of Sport and Health Sciences, GIH, Stockholm, Sweden

* Corresponding author
E-mail: peter.schantz@gih.se

Table S1 Measured $\dot{V}O_2$, HR, $\dot{V}O_2$/HR and RPE for submaximal and maximal ergometer cycling

	Workload	$\dot{V}O_2$			HR			$\dot{V}O_2$/HR	RPE	
	W	$L \cdot min^{-1}$	$mL \cdot min^{-1} \cdot kg^{-1}$	%$\dot{V}O_2$max cycling	$beats \cdot min^{-1}$	%HRmax cycling	%HRR cycling	$L \cdot min^{-1}$/ $beats \cdot min^{-1}$	legs	breathing
**Women**	**50**	0.85 ± 0.07	14.2 ± 1.4	30.4 ± 2.5	92.2 ± 11.4	51.5 ± 4.7	32.8 ± 5.5	0.0093 ± 0.0012	9.1 ± 1.8	8.8 ± 1.8
n = 12	**75**	1.13 ± 0.07	18.9 ± 1.3	40.6 ± 3.4	106.5 ± 13.6	59.4 ± 5.7	43.9 ± 6.5	0.0108 ± 0.0013	10.8 ± 1.9	10.6 ± 1.9
BW: (kg)	**100**	1.44 ± 0.06	24.1 ± 2.0	51.9 ± 5.5	121.5 ± 15.7	67.8 ± 6.5	55.5 ± 7.7	0.0120 ± 0.0013	12.7 ± 1.2	12.2 ± 1.3
60.3 ± 5.0	**125**	1.78 ± 0.08	29.7 ± 2.7	64.0 ± 7.2	138.2 ± 17.1	77.1 ± 6.8	68.4 ± 8.6	0.0130 ± 0.0012	14.3 ± 1.1	13.4 ± 1.4
	**150**	2.09 ± 0.08	34.8 ± 2.9	75.0 ± 7.7	150.4 ± 17.1	84.0 ± 6.7	77.9 ± 8.6	0.0140 ± 0.0012	15.8 ± 0.9	15.0 ± 1.1
	**Max**	2.81 ± 0.26	46.7 ± 4.3	100.0 ± 0.0	178.9 ± 10.7	100.0 ± 0.0	100.0 ± 0.0	0.0158 ± 0.0018	19.2 ± 0.6	18.4 ± 0.8
**Men**	**50**	1.06 ± 0.07	13.1 ± 1.5	24.1 ± 2.5	83.5 ± 10.6	46.7 ± 4.4	25.2 ± 5.5	0.0128 ± 0.0018	7.6 ± 1.1	7.5 ± 1.2
n = 12	**75**	1.29 ± 0.10	16.0 ± 2.0	29.4 ± 3.1	91.7 ± 12.5	51.3 ± 5.2	31.6 ± 6.9	0.0143 ± 0.0021	8.8 ± 1.0	8.3 ± 1.4
BW: (kg)	**100**	1.58 ± 0.10	19.7 ± 2.7	36.2 ± 4.0	100.8 ± 13.3	56.3 ± 5.3	38.7 ± 7.3	0.0159 ± 0.0021	10.2 ± 1.3	9.3 ± 1.2
81.2 ± 9.0	**125**	1.88 ± 0.10	23.4 ± 2.9	42.9 ± 4.5	108.6 ± 13.7	60.7 ± 5.2	44.8 ± 7.2	0.0175 ± 0.0024	11.2 ± 1.5	10.4 ± 1.4
	**150**	2.18 ± 0.09	27.2 ± 3.7	49.9 ± 5.9	118.3 ± 15.3	66.1 ± 6.0	52.4 ± 8.5	0.0187 ± 0.0024	12.7 ± 1.2	11.9 ± 1.4
	**Max**	4.42 ± 0.56	54.9 ± 8.0	100.0 ± 0.0	178.7 ± 11.4	100.0 ± 0.0	100.0 ± 0.0	0.0248 ± 0.0033	19.1 ± 0.8	19.1 ± 0.9
**All**	**50**	0.95 ± 0.12	13.6 ± 1.5	27.3 ± 4.0	87.8 ± 11.6	49.1 ± 5.1	29.0 ± 6.6	0.0111 ± 0.0023	8.3 ± 1.7	8.1 ± 1.7
n = 24	**75**	1.21 ± 0.11	17.4 ± 2.2	35.0 ± 6.5	99.1 ± 14.8	55.3 ± 6.8	37.8 ± 9.1	0.0125 ± 0.0025	9.8 ± 1.8	9.4 ± 2.0
BW: (kg)	**100**	1.51 ± 0.11	21.9 ± 3.2	44.0 ± 9.3	111.2 ± 17.7	62.1 ± 8.2	47.1 ± 11.3	0.0140 ± 0.0026	11.4 ± 1.8	10.8 ± 1.9
70.8 ± 12.8	**125**	1.83 ± 0.10	26.5 ± 4.2	53.5 ± 12.3	123.4 ± 21.4	68.9 ± 10.3	56.6 ± 14.3	0.0153 ± 0.0030	12.8 ± 2.0	11.9 ± 2.1
	**150**	2.13 ± 0.10	31.0 ± 5.1	62.5 ± 14.5	134.4 ± 22.8	75.0 ± 11.1	65.2 ± 15.5	0.0163 ± 0.0030	14.2 ± 1.9	13.5 ± 2.0
	**Max**	3.62 ± 0.93	50.8 ± 7.5	100.0 ± 0.0	178.8 ± 10.8	100.0 ± 0.0	100.0 ± 0.0	0.0203 ± 0.0053	19.1 ± 0.7	18.8 ± 0.9

Values are presented as mean ± SD.
n = number of participants, and BW = Body weight.

Table S2 Measured $\dot{V}O_2$, HR, $\dot{V}O_2$/HR and RPE for submaximal treadmill walking

	Workload	$\dot{V}O_2$			HR			$\dot{V}O_2$/HR	RPE	
	**km·h^{-1}**	**L·min^{-1}**	**mL·min^{-1} ·kg^{-1}**	**%$\dot{V}O_2$max running**	**beats·min^{-1}**	**%HRmax running**	**%HRR running**	**L·min^{-1}/ beats·min^{-1}**	**legs**	**breathing**
**Women**	**3**	0.50 ± 0.08	8.3 ± 1.2	16.4 ± 2.2	75.6 ± 9.3	41.0 ± 5.0	19.1 ± 7.1	0.0066 ± 0.0010	7.0 ± 0.7	7.2 ± 0.9
n = 12	**4**	0.59 ± 0.08	9.9 ± 1.2	19.6 ± 2.4	79.9 ± 9.8	43.2 ± 5.1	22.2 ± 6.9	0.0075 ± 0.0009	7.9 ± 1.2	7.9 ± 1.6
BW: (kg)	**5**	0.72 ± 0.09	12.0 ± 1.3	23.9 ± 2.5	86.0 ± 10.4	46.5 ± 5.3	26.7 ± 7.4	0.0085 ± 0.0009	8.8 ± 1.2	8.9 ± 1.5
60.2 ± 5.1	**6**	0.91 ± 0.11	15.1 ± 1.4	30.1 ± 3.4	93.9 ± 11.7	50.8 ± 5.7	32.6 ± 7.9	0.0097 ± 0.0009	10.3 ± 1.2	10.5 ± 1.7
	**7**	1.21 ± 0.12	20.1 ± 1.3	40.0 ± 4.0	107.4 ± 11.9	58.1 ± 5.5	42.5 ± 7.9	0.0113 ± 0.0009	11.8 ± 1.8	11.6 ± 1.8
**Men**	**3**	0.75 ± 0.07	9.2 ± 0.6	15.8 ± 1.3	69.1 ± 5.5	37.4 ± 2.6	13.2 ± 4.5	0.0109 ± 0.0016	6.6 ± 0.5	6.4 ± 0.7
n = 12	**4**	0.85 ± 0.08	10.5 ± 0.8	17.9 ± 1.7	73.7 ± 6.0	39.9 ± 3.1	16.7 ± 4.9	0.0116 ± 0.0015	6.9 ± 0.9	7.1 ± 1.4
BW: (kg)	**5**	1.00 ± 0.09	12.3 ± 0.7	21.1 ± 2.0	78.5 ± 6.3	42.5 ± 3.1	20.3 ± 4.7	0.0128 ± 0.0017	7.8 ± 1.4	7.7 ± 1.6
81.4 ± 9.1	**6**	1.24 ± 0.08	15.4 ± 1.3	26.2 ± 1.8	85.3 ± 6.1	46.2 ± 2.8	25.4 ± 4.3	0.0146 ± 0.0017	8.8 ± 1.9	8.6 ± 1.8
	**7**	1.61 ± 0.13	19.9 ± 1.6	34.0 ± 3.0	95.3 ± 7.2	51.5 ± 3.2	32.9 ± 4.7	0.0170 ± 0.0023	10.2 ± 1.9	9.6 ± 1.7
**All**	**3**	0.62 ± 0.15	8.7 ± 1.1	16.1 ± 1.8	72.3 ± 8.2	39.2 ± 4.3	16.2 ± 6.5	0.0088 ± 0.0026	6.8 ± 0.7	6.8 ± 0.9
n = 24	**4**	0.72 ± 0.15	10.2 ± 1.1	18.8 ± 2.2	76.8 ± 8.6	41.5 ± 4.5	19.5 ± 6.5	0.0095 ± 0.0024	7.4 ± 1.1	7.5 ± 1.5
BW: (kg)	**5**	0.86 ± 0.16	12.2 ± 1.0	22.5 ± 2.6	82.2 ± 9.2	44.5 ± 4.7	23.5 ± 6.9	0.0106 ± 0.0026	8.3 ± 1.4	8.3 ± 1.7
70.8 ± 13.0	**6**	1.08 ± 0.19	15.2 ± 1.4	28.1 ± 3.3	89.6 ± 10.1	48.5 ± 5.0	29.0 ± 7.2	0.0122 ± 0.0028	9.6 ± 1.8	9.5 ± 2.0
	**7**	1.41 ± 0.24	20.0 ± 1.5	37.0 ± 4.6	101.3 ± 11.4	54.8 ± 5.5	37.7 ± 8.1	0.0142 ± 0.0034	11.0 ± 2.0	10.6 ± 2.0

Values are presented as mean ± SD.
n = number of participants, and BW = Body weight.

Table S3 Measured V̇O$_2$, HR, V̇O$_2$/HR and RPE for submaximal and maximal treadmill running

	Workload	V̇O$_2$			HR			V̇O$_2$/HR	RPE	
	km·h^{-1}	L·min^{-1}	mL·min^{-1} ·kg^{-1}	%V̇O$_2$max running	beats·min^{-1}	%HRmax running	%HRR running	L·min^{-1}/ beats·min^{-1}	legs	breathing
**Women**	**6**	1.38 ± 0.13	22.9 ± 1.7	45.7 ± 5.0	112.1 ± 12.8	60.6 ± 5.4	46.1 ± 6.5	0.0124 ± 0.0013	8.4 ± 1.8	8.5 ± 2.0
n = 12	**7**	1.54 ± 0.13	25.6 ± 1.3	51.0 ± 4.1	122.7 ± 13.1	66.3 ± 5.0	54.0 ± 6.2	0.0127 ± 0.0013	9.0 ± 1.8	9.6 ± 2.2
BW: (kg)	**8**	1.71 ± 0.15	28.4 ± 1.2	56.6 ± 4.1	131.5 ± 12.2	71.1 ± 4.5	60.5 ± 5.3	0.0131 ± 0.0014	10.8 ± 1.7	10.9 ± 1.7
60.3 ± 5.0	**10**	2.04 ± 0.15	33.9 ± 1.5	67.4 ± 5.3	146.9 ± 12.4	79.5 ± 4.3	72.0 ± 5.2	0.0139 ± 0.0013	12.6 ± 1.7	12.8 ± 1.7
	**12**	2.43 ± 0.19	40.3 ± 1.7	80.4 ± 6.6	163.8 ± 12.4	88.6 ± 3.9	84.5 ± 5.0	0.0149 ± 0.0011	14.8 ± 1.1	15.3 ± 1.2
	**Max**	3.03 ± 0.27	50.4 ± 3.5	100.0 ± 0.0	184.8 ± 9.5	100.0 ± 0.0	100.0 ± 0.0	0.0164 ± 0.0014	18.5 ± 0.9	19.1 ± 0.5
**Men**	**6**	1.86 ± 0.24	23.0 ± 1.8	39.2 ± 3.7	103.3 ± 10.0	55.8 ± 4.2	38.9 ± 4.6	0.0182 ± 0.0029	7.4 ± 0.8	7.1 ± 0.5
n = 12	**7**	2.12 ± 0.24	26.3 ± 2.2	44.8 ± 3.7	114.4 ± 11.1	61.8 ± 4.5	47.2 ± 5.2	0.0187 ± 0.0027	8.3 ± 1.3	8.3 ± 1.1
BW: (kg)	**8**	2.37 ± 0.28	29.4 ± 2.7	50.0 ± 4.9	122.2 ± 11.1	66.1 ± 4.7	53.1 ± 5.7	0.0195 ± 0.0028	10.0 ± 1.7	9.3 ± 1.4
81.0 ± 9.3	**10**	2.82 ± 0.33	34.9 ± 3.0	59.4 ± 5.4	136.3 ± 10.8	73.7 ± 4.7	63.6 ± 6.0	0.0208 ± 0.0027	11.8 ± 1.6	11.7 ± 1.4
	**12**	3.33 ± 0.37	41.2 ± 3.4	70.0 ± 4.6	151.9 ± 10.8	82.1 ± 4.5	75.4 ± 5.9	0.0220 ± 0.0027	13.8 ± 0.8	13.8 ± 1.3
	**Max**	4.76 ± 0.52	59.1 ± 5.9	100.0 ± 0.0	185.0 ± 9.1	100.0 ± 0.0	100.0 ± 0.0	0.0258 ± 0.0033	18.3 ± 1.2	19.0 ± 0.9
**All**	**6**	1.62 ± 0.31	23.0 ± 1.7	42.5 ± 5.4	107.7 ± 12.1	58.2 ± 5.3	42.5 ± 6.6	0.0153 ± 0.0037	7.9 ± 1.5	7.8 ± 1.6
n = 24	**7**	1.83 ± 0.35	26.0 ± 1.8	47.9 ± 5.0	118.6 ± 12.6	64.1 ± 5.2	50.6 ± 6.6	0.0157 ± 0.0037	8.7 ± 1.6	8.9 ± 1.8
BW: (kg)	**8**	2.04 ± 0.40	28.9 ± 2.1	53.3 ± 5.5	126.9 ± 12.4	68.6 ± 5.2	56.8 ± 6.6	0.0163 ± 0.0039	10.4 ± 1.7	10.1 ± 1.7
70.6 ± 12.8	**10**	2.43 ± 0.47	34.4 ± 2.4	63.4 ± 6.7	141.6 ± 12.6	76.6 ± 5.3	67.8 ± 7.0	0.0174 ± 0.0041	12.2 ± 1.7	12.3 ± 1.6
	**12**	2.88 ± 0.54	40.8 ± 2.7	75.2 ± 7.7	157.9 ± 12.9	85.4 ± 5.3	79.9 ± 7.1	0.0184 ± 0.0042	14.3 ± 1.1	14.6 ± 1.4
	**Max**	3.90 ± 0.97	54.8 ± 6.5	100.0 ± 0.0	184.9 ± 9.1	100.0 ± 0.0	100.0 ± 0.0	0.0211 ± 0.0054	18.4 ± 1.1	19.0 ± 0.7

Values are presented as mean ± SD.
n = number of participants, and BW = Body weight.
