# Supplementary material for: Interchangeability and optimization of heart rate methods for estimating oxygen uptake in ergometer cycling, level treadmill walking and running
Source: BMC Med Res Methodol. 2022 Feb 27;22:55. doi: 10.1186/s12874-022-01524-w (PMC8883654; doi:10.1186/s12874-022-01524-w)
Supplement: Supplementary file 2 — Additional file 2: Table S4 Regression equations of model 1 and exercise mode comparisons. Table S5 Regression equations of model 2 and exercise mode comparisons. [file 12874_2022_1524_MOESM2_ESM.pdf]

Additional file 2

To:
Interchangeability and optimization of heart rate methods for estimating oxygen uptake in ergometer cycling, level treadmill walking and running

BMC Medical Research Methodology

Karin Sofia Elisabeth Olsson[1], Hans Rosdahl[2] & Peter Schantz[1]*

[1]The Research Unit for Movement, Health and Environment, Department of Physical Activity and Health, The Swedish School of Sport and Health Sciences, GIH, Stockholm, Sweden, [2]The Research Unit for Movement, Health and Environment, Department of Physiology, Nutrition and Biomechanics, The Swedish School of Sport and Health Sciences, GIH, Stockholm, Sweden

* Corresponding author
E-mail: peter.schantz@gih.se

Table S4 Regression equations of model 1 and exercise mode comparisons

		y-intercept	slope	r^2
**Coefficients**	**Cycling**[a]	-1.44 ± 0.54 (-1.67 to -1.21)	0.0276 ± 0.0084 (0.0240 to 0.0312)	0.992 ± 0.006 (0.989 to 0.995)
	**Walking**[b]	-1.43 ± 0.42 (-1.61 to -1.25)	0.0285 ± 0.0073 (0.0254 to 0.0315)	0.987 ± 0.010 (0.983 to 0.991)
	**Running**[c]	-1.16 ± 0.47 (-1.36 to -0.96)	0.0256 ± 0.0059 (0.0231 to 0.0281)	0.988 ± 0.010 (0.984 to 0.993)
**Absolute differences**	**Walk *vs* Cyc**	0.01 ± 0.50 (-0.20 to 0.22)	0.0009 ± 0.0053 (-0.0014 to 0.0031)	-0.005 ± 0.012 (-0.010 to 0.000)
	**Run *vs* Cyc**	0.28 ± 0.52 (0.06 to 0.50)*	-0.0020 ± 0.0050 (-0.0041 to 0.0001)	-0.004 ± 0.011 (-0.008 to 0.001)
	**Run *vs* Walk**	0.27 ± 0.63 (0.01 to 0.54)	-0.0028 ± 0.0057 (-0.0052 to -0.0004)*	0.001 ± 0.014 (-0.005 to 0.007)
**Relative differences (%)**	**Walk *vs* Cyc**	5.5 ± 30.3 (-7.3 to 18.2)	5.1 ± 16.5 (-1.8 to 12.1)	-0.5 ± 1.2 (-1.0 to 0.0)
	**Run *vs* Cyc**	-15.7 ± 29.0 (-27.9 to -3.5)*	-4.9 ± 12.9 (-10.3 to 0.5)	-0.4 ± 1.1 (-0.8 to 0.1)
	**Run *vs* Walk**	-12.4 ± 41.0 (-29.7 to 4.9)	-7.4 ± 18.8 (-15.3 to 0.6)	0.1 ± 1.5 (-0.5 to 0.8)

Values are based on the individual measured values (Additional file 1; Table S1-S3) and presented as mean ± SD and (95% CI) (n = 24).
Significance of exercise mode differences: *P < 0.05.
Calculation of the absolute differences: Walk vs Cyc = b-a, Run vs Cyc = c-a and Walk vs Run = c-b.
Calculation of the relative differences: Walk vs Cyc = $((b-a) \cdot a^{-1}) \cdot 100$, Run vs Cyc = $((c-a) \cdot a^{-1}) \cdot 100$ and Walk vs Run = $((c-b) \cdot b^{-1}) \cdot 100$.

Table S5 Regression equations of model 2 and exercise mode comparisons

		y-intercept	slope	r^2
**Coefficients**	**Cycling**[a]	-1.61 ± 0.54 (-1.84 to -1.38)	0.0290 ± 0.0075 (0.0259 to 0.0322)	0.992 ± 0.008 (0.989 to 0.996)
	**Walking**[b]	-1.51 ± 0.39 (-1.67 to -1.34)	0.0292 ± 0.0070 (0.0263 to 0.0322)	0.998 ± 0.002 (0.997 to 0.998)
	**Running**[c]	-1.61 ± 0.64 (-1.87 to -1.34)	0.0292 ± 0.0075 (0.0261 to 0.0324)	0.985 ± 0.011 (0.980 to 0.989)
**Absolute differences**	**Walk *vs* Cyc**	0.10 ± 0.42 (-0.08 to 0.28)	0.0002 ± 0.0027 (-0.0009 to 0.0013)	0.005 ± 0.009 (0.002 to 0.009)*
	**Run *vs* Cyc**	0.00 ± 0.42 (-0.17 to 0.18)	0.0002 ± 0.0028 (-0.0010 to 0.0014)	-0.008 ± 0.011 (-0.012 to -0.003)**
	**Run *vs* Walk**	-0.10 ± 0.47 (-0.30 to 0.10)	-0.0000 ± 0.0025 (-0.0011 to 0.0010)	-0.013 ± 0.012 (-0.018 to -0.008)***
**Relative differences (%)**	**Walk *vs* Cyc**	-1.5 ± 24.0 (-11.6 to 8.6)	1.7 ± 9.5 (-2.3 to 5.7)	0.5 ± 0.9 (0.2 to 0.9)*
	**Run *vs* Cyc**	1.3 ± 28.3 (-10.6 to 13.2)	1.3 ± 10.1 (-3.0 to 5.5)	-0.8 ± 1.1 (-1.2 to -0.3)**
	**Run *vs* Walk**	7.1 ± 32.7 (-6.7 to 20.9)	-0.1 ± 8.9 (-3.8 to 3.7)	-1.3 ± 1.2 (-1.8 to -0.8)***

Values are based on the individual measured values (Additional file 1; Table S1-S3) and presented as mean ± SD and (95% CI) (n = 24).
Significance of exercise mode differences: *P < 0.05, **P < 0.01, ***P < 0.001.
Calculation of the absolute differences: Walk vs Cyc = b-a, Run vs Cyc = c-a and Walk vs Run = c-b.
Calculation of the relative differences: Walk vs Cyc = $((b-a) \cdot a^{-1}) \cdot 100$, Run vs Cyc = $((c-a) \cdot a^{-1}) \cdot 100$ and Walk vs Run = $((c-b) \cdot b^{-1}) \cdot 100$.
