# Supplementary material for: Interchangeability and optimization of heart rate methods for estimating oxygen uptake in ergometer cycling, level treadmill walking and running
Source: BMC Med Res Methodol. 2022 Feb 27;22:55. doi: 10.1186/s12874-022-01524-w (PMC8883654; doi:10.1186/s12874-022-01524-w)
Supplement: Supplementary file 3 — Additional file 3: A comparative analysis with the aim of optimizing the HR method: Table S6 Linear regression equations for the exercise mode comparisons of the estimated individual V̇O2 values in Olsson, Salier Eriksson (1) and the present study. Table S7 Linear regression equations for the model comparisons of the estimated individual V̇O2 values in Olsson, Salier Eriksson (1) and the present study. Figure S1. The average HR-V̇O2 measurement ranges in Olsson, Salier Eriksson (1) and the present study. Figure S2. Exercise mode comparisons for the estimated individual V̇O2 values in Olsson, Salier Eriksson (1). Figure S3. Exercise mode comparisons for the estimated individual V̇O2 values in the present study. Figure S4. Model comparisons for the estimated individual V̇O2 values in Olsson, Salier Eriksson (1). Figure S5. Model comparisons for the estimated individual V̇O2 values in the present study. [file 12874_2022_1524_MOESM3_ESM.pdf]

Additional file 3

To:
Interchangeability and optimization of heart rate methods for estimating oxygen uptake in ergometer cycling, level treadmill walking and running

BMC Medical Research Methodology

Karin Sofia Elisabeth Olsson[1], Hans Rosdahl[2] & Peter Schantz[1]*

[1]The Research Unit for Movement, Health and Environment, Department of Physical Activity and Health, The Swedish School of Sport and Health Sciences, GIH, Stockholm, Sweden, [2]The Research Unit for Movement, Health and Environment, Department of Physiology, Nutrition and Biomechanics, The Swedish School of Sport and Health Sciences, GIH, Stockholm, Sweden

* Corresponding author
E-mail: peter.schantz@gih.se

A comparative analysis with the aim of optimizing the HR method

Abstract

Introduction: Heart rate (HR) methods enable estimations of oxygen uptake ($\dot{V}O_2$) in physical activities. It has been shown that varying the ways of establishing the HR-$\dot{V}O_2$ relationships, as part of HR methods, lead to different degrees of methodological optimization. This analysis therefore aims to evaluate the effects of using different numbers of workloads and widths of the measurement ranges when establishing HR-$\dot{V}O_2$ relationships.

Methods: Comparisons of HR methods applied to ergometer cycling and level treadmill walking have been made between the present study (n = 24) and a previous (n = 34). Both studies examined two different models of HR-$\dot{V}O_2$ relationships. Model 1 was based on only submaximal workloads, whereas model 2 also included a workload of maximal exercise. Three submaximal workloads were used in the previous study, while the present study included five loads, and a wider range for walking.

Results: The individual $\dot{V}O_2$ variations in the comparisons of walking and cycling, with both model 1 and model 2, were lower when using more submaximal workloads and a wider measurement range for walking. When comparing model 1 and model 2 for walking, the individual $\dot{V}O_2$ variations were reduced with more submaximal workloads and a wider measurement range. No such differences were noted for cycling.

Discussion and Conclusion: The comparisons between the two studies are dependent on two different samples of participants. Therefore we cannot rule out that the observed differences may, at least partly, be due to varying group characteristics rather than to the differences in the HR methods applied. However, at this stage of analysis we conclude that more submaximal workloads and wider ranges enhance the interchangeability between cycling and walking, and optimize the HR methods.

Introduction

Applying heart rate (HR) methods for estimating oxygen uptake ($\dot{V}O_2$) during physical activities can be of value for education, health promotion and disease prevention as well as research purposes. For these purposes, the HR methods need to be methodologically developed. In this respect, a previous study (1) examined the interchangeability of HR methods between ergometer cycling and level treadmill walking. It found that the individual variation, when applying the HR methods, was reduced when the HR-$\dot{V}O_2$ relationships were based on both submaximal and maximal workloads as compared to only submaximal. In this analysis, we will instead focus on evaluating the effects of using more submaximal workloads (five *vs* three) as well as wider measurement ranges when establishing the HR-$\dot{V}O_2$ relationships. This is done through comparing results between the present study and Olsson, Salier Eriksson (1).

Methods

Systematic comparisons of HR methods applied to ergometer cycling and level treadmill walking have been made between the present study and a previous one (1). In the present study, 24 participants (12 males and 12 females; age 29 ± 8 years) were included, whereas 34 participants (17 males and 17 females; age 44 ± 6 years) participated in Olsson, Salier Eriksson (1). For further information of the different groups of participants, see the Methods sections in the present study and in Olsson, Salier Eriksson (1).

In both studies, two different models of establishing HR-$\dot{V}O_2$ relationships for cycling and walking, respectively, were examined. Model 1 consisted of only submaximal workloads, while model 2 included also a workload of maximal exercise. Three submaximal workloads were used in Olsson, Salier Eriksson (1), whereas the present study included five submaximal workloads, and a clearly wider range for walking. The different HR and $\dot{V}O_2$ measurement ranges are illustrated in Fig. S1. For further procedures and materials used, see the Methods sections in the present study and in Olsson, Salier Eriksson (1).

To perform this comparative analysis, data in Olsson, Salier Eriksson (1) have been recalculated to be comparable with the present study. Furthermore, all illustrations of individual $\dot{V}O_2$ data in Olsson, Salier Eriksson (1), have been redesigned to be comparable with the figures in the present study (cf. Fig. S2-S5).

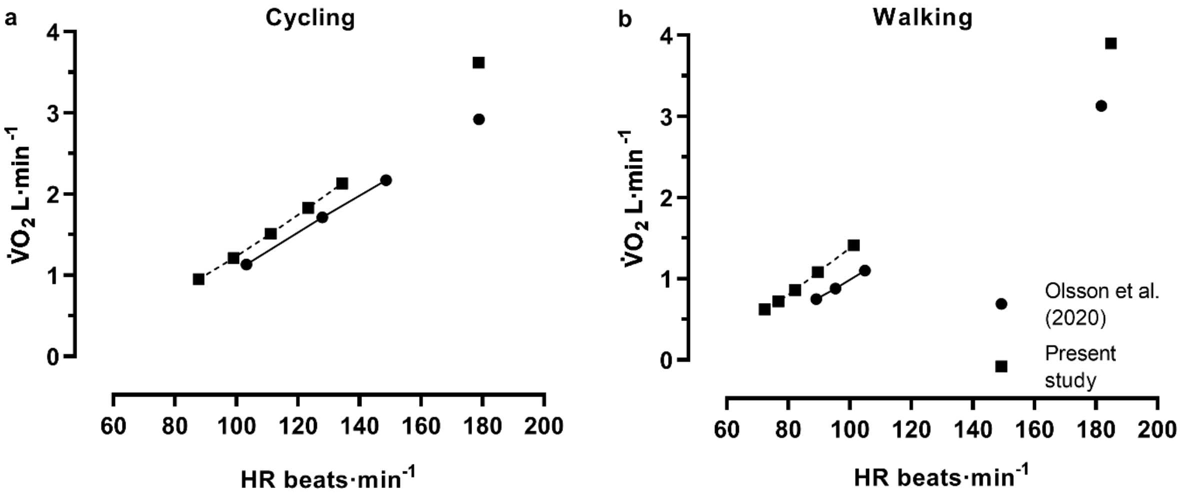

Fig. S1 The average HR-V̇O_2 measurement ranges in Olsson, Salier Eriksson (1) and the present study Submaximal positions (symbols with connected lines) were used to establish the HR-V̇O_2 relationships for both model 1 and model 2, while maximal positions (separated symbols) were used only for model 2. The figure is based on the individual values of all participants in Olsson, Salier Eriksson (1) (n = 34) and the present study (n = 24). (**a**) Submaximal and maximal cycling and (**b**) submaximal walking and maximal running.

Results

Optimization of interchangeability between the exercise modalities

The present comparisons of the estimated individual V̇O_2 values of walking *vs* cycling indicate that there is a reduced individual variation in both models compared to Olsson, Salier Eriksson (1) (Table S6; Fig. S2 and S3). This is based on numerically: (1) higher r^2-coefficients, and (2) narrower 95% confidence intervals for both y-intercepts and slopes in the present study. At the same time, the y-intercepts were numerically closer to y = 0 in Olsson, Salier Eriksson (1) than in the present study. Finally, only minor differences were observed when comparing the slopes between the two studies, and they were all close to the lines of identity (Table S6).

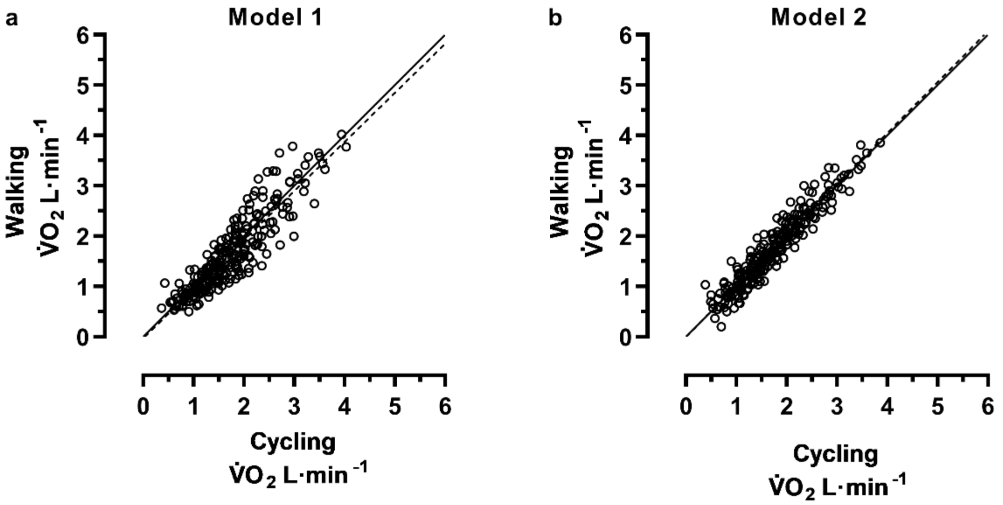

Fig. S2 Exercise mode comparisons for the estimated individual $\dot{V}O_2$ values in Olsson, Salier Eriksson (1)
The figure is based on the individual $\dot{V}O_2$ values of all participants (n = 34), ranging between 25-85 %HRR. Line of identity = solid line and the linear regression = dashed line. Walking *vs* cycling; (**a**) Model 1 and (**b**) model 2. For regression equations and r^2-coefficients, see Table S6.

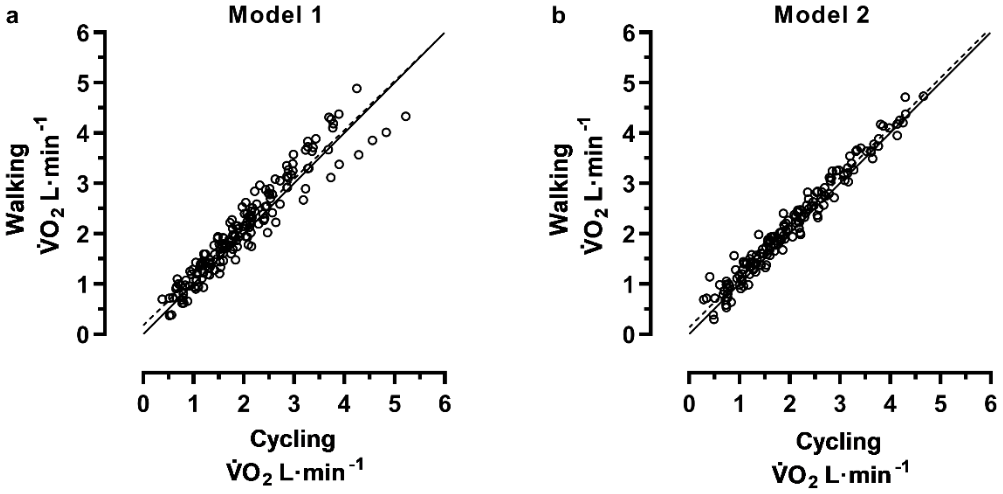

Fig. S3 Exercise mode comparisons for the estimated individual $\dot{V}O_2$ values in the present study
The figure is based on the individual $\dot{V}O_2$ values of all participants (n = 24), ranging between 25-85 %HRR. Line of identity = solid line and the linear regression = dashed line. Walking *vs* cycling; (**a**) Model 1 and (**b**) model 2. For regression equations and r^2-coefficients, see Table S6.

Table S6 Linear regression equations for the exercise mode comparisons of the estimated individual $\dot{V}O_2$ values in Olsson, Salier Eriksson (1) and the present study

		y-intercept (95% CI)	slope (95% CI)	r^2
**Olsson, Salier Eriksson (1)**	Model 1: Walk & Cyc	-0.033 (-0.148 to 0.082)	0.975 (0.914 to 1.036)	0.808
	Model 2: Walk & Cyc	-0.004 (-0.081 to 0.073)	1.013 (0.971 to 1.054)	0.909
**Present study**	Model 1: Walk & Cyc	0.178 (0.077 to 0.279)	0.970 (0.924 to 1.016)	0.913
	Model 2: Walk & Cyc	0.138 (0.073 to 0.202)	0.992 (0.963 to 1.021)	0.965

The linear regression equations are based on the individual $\dot{V}O_2$ values of all participants in Olsson, Salier Eriksson (1) (n = 34) and the present study (n = 24), ranging between 25-85 %HRR (cf. Fig. S2 and S3). Walk = walking and Cyc = cycling.

Optimization within each exercise modality

In the model comparison of individual $\dot{V}O_2$ values, a high level of resemblance along the line of identity was observed for cycling in Olsson, Salier Eriksson (1), and it did not improve in the present study (Table S7; cf. Fig. S4a and S5a). On the other hand, the corresponding model comparison for walking showed a higher resemblance in the present study compared to Olsson, Salier Eriksson (1) (cf. Fig. S4b and S5b). This was indicated by numerically; (1) higher r^2-coefficient, (2) y-intercept closer to y = 0, (3) slope closer to the line of identity, and (4) narrower 95% confidence intervals for both y-intercept and slope (Table S7).

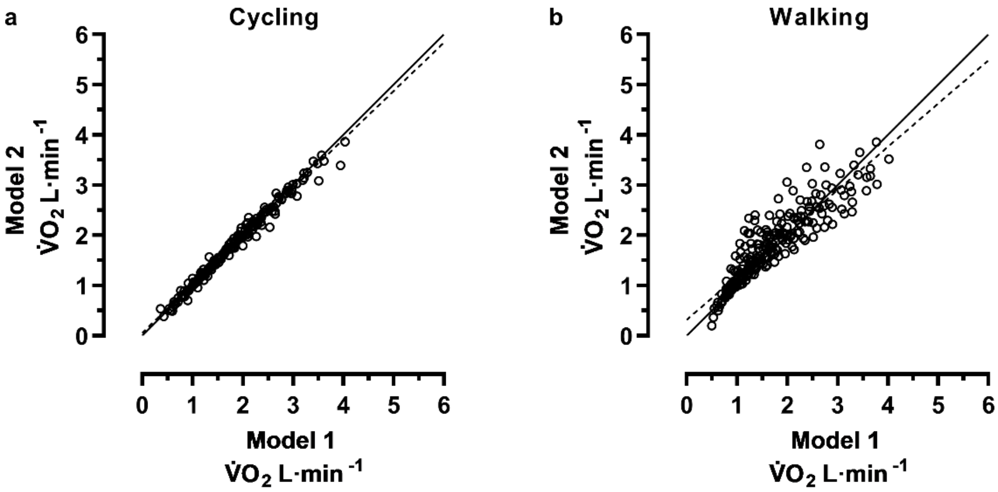

Fig. S4 Model comparisons for the estimated individual $\dot{V}O_2$ values in Olsson, Salier Eriksson (1)
The figure is based on the individual $\dot{V}O_2$ values of all participants (n = 34), ranging between 25-85 %HRR. Line of identity = solid line and the linear regression = dashed line. (**a**) Cycling and (**b**) walking. For regression equations and r^2-coefficients, see Table S7.

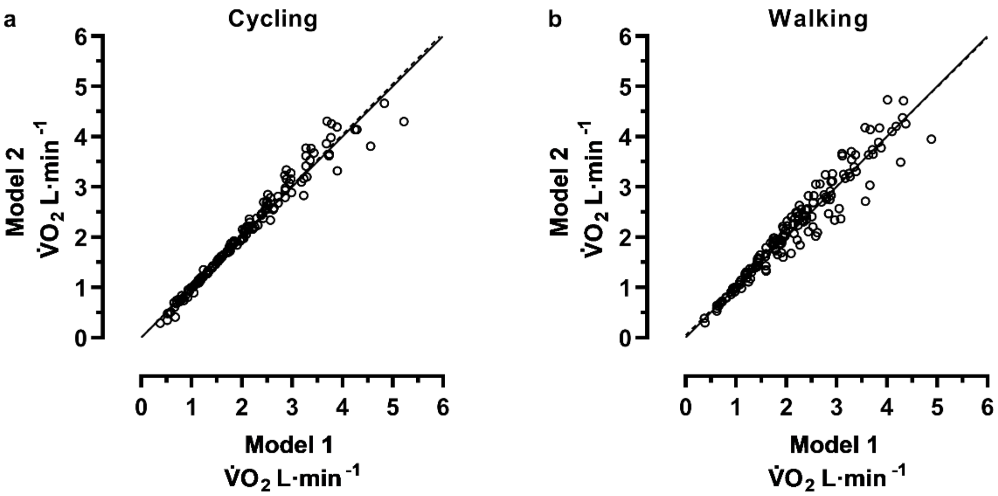

Fig. S5 Model comparisons for the estimated individual $\dot{V}O_2$ values in the present study
The figure is based on the individual $\dot{V}O_2$ values of all participants (n = 24), ranging between 25-85 %HRR. Line of identity = solid line and the linear regression = dashed line. (**a**) Cycling and (**b**) walking. For regression equations and r^2-coefficients, see Table S7.

Table S7 Linear regression equations for the model comparisons of the estimated individual $\dot{V}O_2$ values in Olsson, Salier Eriksson (1) and the present study

		**y-intercept (95% CI)**	**slope (95% CI)**	**r^2**
**Olsson, Salier Eriksson (1)**	Cycling: Model 1 & 2	0.056 (0.028 to 0.084)	0.963 (0.948 to 0.978)	0.986
	Walking: Model 1 & 2	0.318 (0.223 to 0.413)	0.861 (0.809 to 0.912)	0.821
**Present study**	Cycling: Model 1 & 2	0.000 (-0.060 to 0.060)	1.010 (0.983 to 1.037)	0.970
	Walking: Model 1 & 2	0.050 (-0.041 to 0.141)	0.987 (0.948 to 1.026)	0.937

The linear regression equations are based on the individual $\dot{V}O_2$ values of all participants in Olsson, Salier Eriksson (1) (n = 34) and the present study (n = 24), ranging between 25-85 %HRR (cf. Fig. S4 and S5).

Discussion and Conclusion

There were indications of a reduced individual variation in the exercise mode comparisons of walking *vs* cycling (Table S6; Fig. S2 and S3) when additional submaximal workloads (five *vs* three) were used, as well as a wider measurement range for walking. We interpret that this reduction of individual variation enhanced the interchangeability of the HR methods by becoming more accurate.

Given the model comparisons of individual $\dot{V}O_2$ values in cycling and walking, respectively, (Table S7; Fig. S4 and S5), and the fact that the submaximal measurement range for walking in Olsson, Salier Eriksson (1) was almost doubled in the present study (Fig. S1b), there are clear indications that the width of the range is important for the stability of the HR method. Moreover, this indicates that the individual variations between model 1 and model 2 will be reduced when using a wide submaximal range. This decreases the importance of using a maximal workload when establishing HR-$\dot{V}O_2$ relationships and applying HR methods within the same form of exercise.

Finally, note that these comparative analyses of the two studies are based on pairwise numerical comparisons. Due to the low number (two) of possible comparisons, there has been no basis for statistical analyses. Furthermore, the analyses are dependent on two different samples of participants. Therefore we cannot rule out that the observed differences may, at least partly, be due to varying group characteristics rather than to the differences in the HR methods applied. Thus, it would be valuable to also evaluate the issues of optimization strategies through intra-individual comparisons. However, at this stage of analysis, we conclude that more submaximal workloads (five *vs* three) and wider ranges stabilize the HR-$\dot{V}O_2$ relationships and thereby improve the interchangeability between cycling and walking, as well as optimize the HR methods.

Reference

1. Olsson K, Salier Eriksson J, Rosdahl H, Schantz P. Are heart rate methods based on ergometer cycling and level treadmill walking interchangeable? PLOS ONE. 2020;15(8):e0237388.
